# Bimetallic Copper–Manganese Zeolitic Imidazolate Framework Nanozyme Scavenges Reactive Oxygen Species to Alleviate Osteoarthritis via Phosphoinositide 3-Kinase/Mammalian Target of Rapamycin Axis and Autophagic Flux Restoration

**DOI:** 10.34133/bmr.0306

**Published:** 2026-01-21

**Authors:** Xiaoyu Zheng, Su Zhao, Shuming Li, Yanli Wang, Jiani Shi, Yufei Qiu, Xutong Wu, Yanping Zhao, Tao Jia, Tianqi Dai

**Affiliations:** ^1^ Department of Anesthesiology, Harbin Medical University Cancer Hospital, Harbin 150001, China.; ^2^ Department of Thoracic Surgery, Harbin Medical University Cancer Hospital, Harbin 150001, China.; ^3^ Department of Rheumatology, The First Affiliated Hospital of Harbin Medical University, Harbin 150001, China.; ^4^ Harbin Medical University, Harbin 150001, China.; ^5^ NHC and CAMS Key Laboratory of Molecular Probe and Targeted Theranostics, Molecular Imaging Research Center (MIRC); Department of Nuclear Medicine, the Fourth Hospital of Harbin Medical University, Harbin 150028, China.

## Abstract

Osteoarthritis (OA) is the fastest-growing cause of physical disability worldwide, yet no therapy currently halts its age-dependent progression. Increasing evidence suggests that reactive oxygen species (ROS) are central drivers of cartilage degradation and OA progression. Therefore, the clearance of ROS is critical for mitigating OA progression and developing effective therapeutic strategies. In this study, we report a bioinspired copper–manganese zeolitic imidazolate framework (CuMn-ZIF) that integrates catalase (CAT) and superoxide dismutase (SOD)-mimetic activities within a single nanoplatform. By simultaneously scavenging H_2_O_2_ and superoxide anions, the CuMn-ZIF nanozyme rebalances redox status in human OA chondrocytes, suppressing PI3K–AKT–mTOR signaling and restoring lysosomal–autophagic flux. An intra-articular injection in destabilized medial meniscus (DMM) mice markedly ameliorated cartilage deterioration and subchondral bone loss, showing a 1.5-fold increase in bone mineral density (BMD), a 2.1-fold greater bone volume/tissue volume (BV/TV), and a 2-fold increase in trabecular number compared to DMM controls. Comprehensive in vitro and in vivo analyses validated the CuMn-ZIF nanozyme as a potent therapeutic agent, demonstrating exceptional catalytic activity and reproducible disease-modifying effects in OA. This work establishes a scalable blueprint for ROS-targeting, enzyme-mimetic nanomedicines that can potentially be translated to treat OA and other ROS-dependent diseases.

## Introduction

Osteoarthritis (OA) is a progressive, age-related joint pathology that presents with systemic chondrocyte dysfunction and cellular senescence. OA has been linked to pain and impaired mobility in affected individuals. OA prevalence in the global population aged 40 and older is about 23%, causing significant healthcare challenges and increasing social and economic costs [1–3]. Therefore, effective strategies are needed to alleviate and treat OA. Inflammatory mediators in OA patients are often elevated within the joint cavity. Oxidative stress within this environment generates reactive oxygen species (ROS), which can exacerbate inflammation in chondrocytes, induce metabolic dysregulation, and degrade cartilage. Effective control of ROS is crucial for OA treatment, particularly concerning the cartilage [4,5]. Currently, therapeutic strategies for OA predominantly rely on nonsteroidal anti-inflammatory drugs (NSAIDs) and other medications to manage symptoms, which do not address the root cause of the disease [6]. Given the critical role of ROS in the pathophysiology of OA, regulating the complex mechanochemical coupling environment through ROS elimination represents a prospective therapeutic approach.

Antioxidant enzymes, principally superoxide dismutase (SOD) and catalase (CAT), constitute a critical enzymatic defense mechanism for ROS neutralization and cellular redox equilibrium maintenance [7,8]. Emerging clinical evidence indicates substantial down-regulation of these protective enzymes in the pathogenesis of OA, highlighting the potential of biomimetic catalytic strategies as a transformative approach for restoring redox homeostasis in articular pathologies [9]. Biomimetic catalysts have attracted considerable interest in catalytic medicine owing to their potential to mimic natural enzyme functions [10–16]. However, most existing biomimetic catalysts only replicate single active sites [17–19], whereas many natural enzymes operate through multisite synergistic mechanisms.

The future of dual- and multi-site biomimetics thus lies in the rational design of catalysts that replicate the synergistic interactions of multiple active sites. SOD represents a pivotal antioxidant enzyme, functioning as a primary cellular defense mechanism against ROS by neutralizing deleterious superoxide radicals and modulating inflammatory signaling cascades [20]. CuZn-SOD (copper–zinc SOD) serves as a canonical antioxidant enzyme whose catalytic activity derives from its unique histidine-bridged bimetallic active center. Notably, Cu^2+^ is coordinated by 4 histidine residues via Cu–N bonds and one water molecule through a Cu–O bond. Correspondingly, Zn^2+^ is coordinated by 3 histidine residues through Zn–N bonds and one aspartate residue via a Zn–O bond [21,22]. This bimetallic coordination and synergistic interaction mediates its function, underscoring the need for developing dual- or multisite biomimetic systems to more effectively replicate natural enzyme activities [23].

**Fig. 1.**
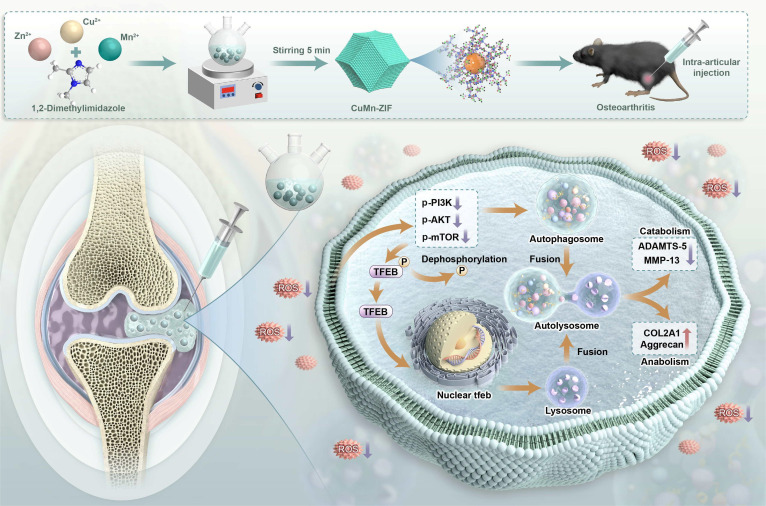
A graphical depiction of the synthesis and ROS scavenging capacity of the CuMn-ZIF nanozyme. The CuMn-ZIF nanozyme enhanced the fusion of autophagosomes and lysosomes by eliminating ROS levels in the joint cavity, promoting autophagic flux, to improve the catabolic and synthetic metabolism levels in OA chondrocytes.

Based on the above understandings, we herein we report the design of a dual-metal biomimetic catalyst, a copper–manganese zeolitic imidazolate framework-based nanozyme (CuMn-ZIF nanozyme), which exhibits a synergistic effect in scavenging ROS and reducing inflammation in OA. CuMn-ZIF nanozyme can directly convert the overexpressed ROS, such as •OH, •O_2_^−^, and H_2_O_2_, to H_2_O and O_2_, due to the SOD and CAT activities, thus reducing ROS levels in cartilage tissue and modulating the phosphoinositide 3-kinase (PI3K)–AKT–mammalian target of rapamycin (mTOR) signaling pathway. Furthermore, CuMn-ZIF nanozyme can improve autophagic flux by enhancing autophagosome and lysosome functions, thereby restoring the metabolic balance between catabolic and anabolic processes in chondrocytes (Fig. 1).

In summary, we proposed to construct a dual-metal biomimetic CuMn-ZIF nanozyme to function as a ROS scavenger, thereby alleviating OA efficiently, validate their protective efficiency of chondrocyte cells under an oxidative stress microenvironment, investigate its underlying molecular mechanisms in vitro, and evaluate their therapeutic efficacy for treating OA in vivo. This innovative strategy holds substantial promise for effectively managing the complex pathology of OA and offers a promising clinical application in the field of OA.

## Materials and Methods

### Preparation of CuMn-ZIF nanozyme

Cu(NO₃)₂•3H₂O (2.4 mg), Mn(NO₃)₂•4H₂O (2.5 mg), and Zn(NO₃)₂•6H₂O (12 mg) were initially dissolved in 0.5 ml of water to form solution A. In a separate vessel, 33 mg of 2-methylimidazole was dissolved in 1 ml of methanol to form solution B. Under continuous stirring, solution A was added dropwise to solution B, and the mixture was allowed to react for 5 min. The resulting mixture was then centrifuged, and the CuMn-ZIF nanozyme was collected by repeated washing with methyl alcohol.

### Reagents and chemicals

Cu(NO₃)₂•3H₂O, Mn(NO₃)₂•4H₂O, Zn(NO₃)₂•6H₂O, 2-methylimidazole, methanol, xanthine, xanthine oxidase, deionized water, acetic acid (HAc)/sodium acetate (NaAc) buffer, 5,5-dimethyl-1-pyrroline N-oxide (DMPO), 2, 2′-azino-bis(3-ethylbenzothiazoline-6-sulfonic acid) (ABTS), protein lysate, and BCA Protein Assay Kit were purchased from Wanleibio. Antibodies were purchased from Cell Signaling Technology (CST), Abcam, Proteintech, Affinity, and Abclonal. Hematoxylin-eosin Mixed Dyeing Solution (One-step method) (G4520) and Millonig Buffered Formalin Fixative (10%) (G2240) were obtained from Solarbio (Beijing Solarbio Science & Technology Co. Ltd.). Safranin-O/Fast Green staining kit was obtained from Beyotime.

### Examination of the H_2_O_2_ scavenging potential of CuMn-ZIF nanozyme

The H_2_O_2_ scavenging capacity of the CuMn-ZIF nanozyme was evaluated using a Hydrogen Peroxide Detection Kit. In this assay, H_2_O_2_ reacts with ammonium molybdate to produce a yellow complex with an absorbance peak at 405 nm. The CuMn-ZIF nanozyme was incubated with 5 mM H_2_O_2_ for 2 h at concentrations of 2 to 12 μg/ml. The residual H_2_O_2_ was measured following the manufacturer’s instructions, and the H_2_O_2_-eliminating capacity was calculated.

### •O_2_^−^ scavenging capacity of CuMn-ZIF nanozyme

A superoxide anion assay kit was employed to detect the scavenging capacity of •O_2_^−^ following the manufacturer’s instructions. Various concentrations of CuMn-ZIF nanozyme (2 to 12 μg/ml) were added to the working solution for 10 min of reaction. The absorbance at 550 nm was quantified utilizing a microplate reader. Electron paramagnetic resonance (EPR) spectroscopy measurements: Superoxide anions were prepared via the reaction between xanthine and xanthine oxidase. A solution of xanthine (0.22 mM, 100 μl) was mixed with a solution of xanthine oxidase (4 units/ml, 5 μl) and 900 μl of deionized water, followed by the addition of varying concentrations of CuMn-ZIF nanozyme (20 μg/ml) in 0.5 M HAc/NaAc buffer (pH 4.5) and 10 mM DMPO. The EPR signals were detected immediately after the addition of the components.

### ABTS radical scavenging capacity of CuMn-ZIF nanozyme

ABTS radicals were prepared by mixing a 7 mM ABTS solution with 2.45 mM potassium persulfate and incubating in the dark for 16 h. The resulting ABTS radical solution was diluted with phosphate-buffered saline (PBS) to an absorbance of ~0.7 at 734 nm. CuMn-ZIF nanozyme solutions (2 to 12 μg/ml) were then mixed with the ABTS solution (2 ml each) and incubated in the dark for 10 min. The absorbance at 734 nm was measured using ultraviolet–visible (UV–Vis) spectroscopy.

### Detection of H_2_O_2_ generation

To detect the generation of H_2_O_2_ by the CuMn-ZIF nanozyme, we conducted chemiluminescence assays. First, we prepared a reaction system containing •O_2_^−^ generated by the xanthine/xanthine oxidase system. The CuMn-ZIF nanozyme was then introduced into this system. Luminol was added as the chemiluminescent probe, and the mixture was placed in a chemiluminescence imaging system to record the intensity of the emitted light. As the reaction progressed and •O_2_^−^ was consumed, a significant increase in chemiluminescent signal intensity was observed, indicating the formation of H_2_O_2_.

### Animal experiments

Mouse modeling: C57/BL6 mice were assigned into 6 groups: Sham group (*n* = 6), destabilized-medial-meniscus (DMM) group (*n* = 6), DMM + ZIF group (*n* = 6), DMM + Cu-ZIF group (*n* = 6), DMM + Mn-ZIF group (*n* = 6), and DMM + CuMn-ZIF group (*n* = 6). The mice underwent destabilization of the medial meniscus (DMM) to establish the OA model, and the mice in the Sham group underwent skin opening and then suturing. The other 2 groups of mice were treated with DMM and intra-jointly injected with ZIF (10 mg/kg), Cu-ZIF (10 mg/kg), Mn-ZIF (10 mg/kg), and CuMn-ZIF nanoparticles (NPs) (10 mg/kg) every other day at 2, 4, 6, and 8 weeks post-surgery.

### Micro-CT

Micro-computed tomography (CT) imaging was performed on the right knee joints of all mice 8 weeks post-surgery to assess the OA changes. The bone histomorphometry parameter analysis included bone mineral density (BMD), trabecular bone volume relative to tissue volume (BV/TV, BVF), trabecular number (Tb.N), trabecular spacing (Tb.Sp), and cortical thickness (Ct.Th).

### In vivo imaging experiments

C57/BL6 mice were anesthetized with an appropriate dose of sodium pentobarbital to minimize stress responses during the imaging procedure. The animals’ body temperature was maintained using a heating pad to ensure stability throughout the imaging process. A high-resolution imaging system, such as a bioluminescence imaging system, was set up and calibrated for the appropriate wavelength or imaging parameters based on the chosen imaging modality. The mice were intravenously injected with L-012 imaging agent. L-012 (10 mg/ml) was prepared in a sterile water solution and Dulbecco’s PBS (DPBS). The mice were placed in the imaging system (IVIS) after 2 min of injection, and images were captured with a 40-s exposure. Data were collected by the imaging system based on bioluminescence signals.

### Safranin-O/Fast Green

The hind limbs of the mice were placed in an EDTA decalcification solution for decalcification for 30 d. The excess ends were removed, and the joints were cut along the sagittal plane. The samples were placed in an automatic dehydrator for tissue dehydration and paraffin embedding*.* Paraffin sections were prepared and stained following the instructions of the modified Safranin-O/Fast Green staining kit (Beyotime, C0621S). Hematoxylin staining was conducted for 10 min and washed off with tap water. The sections were subsequently treated with hydrochloric acid alcohol solution (1% hydrochloric acid, 70% ethanol) for 30 s and rinsed using PBS for 10 min to return to blue. The sections were stained with Fast Green for 5 min and then quickly rinsed using tap water until the cartilage color became colorless. The sections were transferred to acetic acid differentiation solution for 35 s and washed with tap water. The samples underwent Ponceau Red-O staining for 8 min. The samples underwent Ponceau Red-O staining for 8 min and then dehydrated thrice with absolute ethanol, cleared with xylene thrice (5 min each time), and mounted with neutral resin.

### H&E staining

Firstly, the tissue slices were immersed in xylene and the dewaxing treatment was performed with anhydrous ethanol and a series of alcohol solutions, followed by washing with distilled water. The sections were stained with hematoxylin for 5 min to highlight the cell nuclei and then rinsed with distilled water. The sections were briefly differentiated in 1% hydrochloric acid–alcohol solution. The sections were then stained with eosin for 3 min and rinsed with distilled water. The tissue sections underwent a stepwise dehydration process utilizing a series of ethanol concentrations, followed by a clearing procedure involving xylene. Ultimately, the sections were affixed using a neutral resin.

### Immunohistochemical staining

The methodology employed for the preparation of immunohistochemical samples adheres to the protocol established in earlier studies. Initially, the paraffin-embedded sections underwent a series of treatments, including deparaffinization, hydration, antigen retrieval, blocking, and subsequent incubation with antibodies, all facilitated by a paraffin microtome 32. Finally, the sections were mounted for subsequent microscopy imaging and analysis.

### Primary chondrocyte culture and grouping

Chondrocytes were extracted from cartilage via enzymatic digestion, as previously described. The cartilage was extracted and digested with trypsin (SEVEN, SC109-01) for 20 min, followed by overnight digestion with collagenase II (Gibco, 17101015). The digested tissue was filtered and centrifuged through a cell strainer and cultured in medium supplemented with 15% fetal bovine serum (FBS) for 24 h. Subsequently, the cells were assigned to 4 groups: control, H_2_O_2_, H_2_O_2_ + ZIF, and H_2_O_2_ + CuMn-ZIF. The mice were treated with H_2_O_2_ (500 μM), ZIF (2.5 μg/ml), or CuMn-ZIF (3.125 μg/ml) for 24 h, except for mice in the control group. The same grouping and treatments were applied in subsequent experiments.

### Western blotting

Chondrocytes (1 × 10^6^ cells per well) were seeded into culture dishes and incubated overnight. CuMn-ZIF nanozyme (3.125 μg/ml) and ZIF (2.5 μg/ml) were pretreated for 12 h. Cell proteins were extracted using radioimmunoprecipitation assay (RIPA). Protein concentrations were measured using a bicinchoninic acid (BCA) protein assay kit (Rockford, IL, USA). The proteins were separated on a 12.5% gel (Yaem), with markers added at both ends. For protein separation, electrophoresis was conducted at 90 V and the voltage was increased to 150 V. The proteins were transferred onto a nitrocellulose (NC) membrane and treated with blocking solution for 2 h, followed by overnight incubation with primary antibodies at 4 °C : AKT (CST, 9272), p-AKT (CST, 4060S), mTOR (CST, 2983), p-mTOR (CST, 5536), PI3K (Abclonal, A0982), p-PI3K (Abbkine, ABP50495), LC3B (Abcam, ab192890), P62 (Abcam, ab56416), TFEB (CST, 37681), and p-TFEB. glyceraldehyde-3-phosphate dehydrogenase (GAPDH) (Absin, abs830030; Affinit, AF7021) was used as the housekeeping protein. The membranes were rinsed with tris-buffered saline with Tween 20 (TBST) and then incubated with fluorescently labeled secondary antibodies (IRDye 800CW Goat anti-Rabbit IgG, IRDye 700CW Goat anti-Mouse IgG, LI-COR, 1:15,000) for 1 h at room temperature. Protein bands were visualized and quantified using an Odyssey CLx Imaging System (LI-COR Biosciences).

### Immunofluorescence

Chondrocytes(1 × 10^5^ per well) were plated in 12-well plates and maintained until 70% to 80% confluency. The cells were removed from the incubator after 24 h, gently washed twice with PBS buffer (10 s per wash), and fixed with 4% paraformaldehyde (PFA) on ice for 15 min. The cells were cleaned twice with cold PBS buffer and permeabilized with 0.3% Triton at 4 °C for 15 min. The cells were washed 3 times with cold PBS, blocked with goat serum for 30 min, and then incubated with primary antibodies at 4 °C for 24 h. The cells were washed 3 times with PBS buffer. fluorescein isothiocyanate (FITC)-conjugated secondary antibodies (Proteintech, SA00003-2) were applied for 1 h at 37 °C in the dark. The cells were washed with PBS for several times and mounted with 4′,6-diamidino-2-phenylindole (DAPI)-containing fluorescent mounting medium (ZLI-9557, Zhongshan Gold Bridge), and a confocal laser microscope was used for imaging.

### Autophagic flux detection

A dual-labeled autophagy adenovirus [Hanbio Biotechnology, mCherry–green fluorescent protein–microtubule-associated protein 1A/1B–light chain 3 (mCherry-GFP-LC3)] was used to detect autophagic flux. Healthy cell populations were plated in 96-well plates at optimal density for subsequent assays. The virus was gradually thawed on ice, after which the original culture medium was aspirated. Next, 50 μl of medium supplemented with the virus was added. The total volume was adjusted to 100 μl after 4 h. After 24 h, the medium containing the virus was replaced, and the culture was maintained under standard conditions (37 °C, 5% CO₂) for 48 h. Imaging was performed using an immunofluorescence microscope. Yellow signals, marking autophagosomes (mCherry-GFP-LC3), and red signals, indicating mature autolysosomes (after GFP fluorescence quenching in lysosomes), were detected. The number of yellow and red spots after overlay observed following image overlay was manually counted by 3 independent observers. The resulting data were subsequently presented as a bar graph.

### Short-interfering RNA transfection

Chondrocytes were transfected with negative control short-interfering RNA (siRNA) or siRNA against TFEB (si*TFEB*) using RNAi-Mate reagent (GenePharma, Shanghai, China) for 48 h, following the manufacturer’s instructions. Chondrocytes were treated with H_2_O_2_ (500 μM) and CuMn-ZIF (3.125 μg/ml) for 24 h. Cells were used for subsequent experiments.

### LysoTracker staining and detection by FACS

First, the cells were distributed into 6-well plates, and blank control tubes were prepared. The experiment was initiated when the cell density reached 80% to 95%. The cells were rinsed twice using PBS before incubation with LysoTracker dye at 37 °C for 30 min to allow the dye to fully integrate with the lysosomes. The LysoTracker dye (CST, LysoTracker Green DND-26 #8783) was diluted to a final working concentration of 50 nM (1:20,000) before incubation. The cells were trypsinized after washing twice with PBS and analyzed via flow cytometry.

### Cell viability

The cells were digested with trypsin and centrifuged. The collected cells were resuspended in culture medium containing serum and counted using a hemocytometer. The cell suspension was diluted to 3 × 10^4^ cells/ml and seeded into a 96-well plate at 100 μl per well. Once the cells adhered to the plate, drug treatments were performed as detailed above. Each group had 3 replicates, with blank and control groups included. The plate was incubated at 37 °C with 5% CO₂ for 24 h. Following the incubation, 10 μl of Cell Counting Kit-8 (CCK-8) solution was added to each well and the plate was gently agitated. Following an additional 4-h incubation, the absorbance at 450 nm was determined by a microplate reader.

In addition, the cell viability was also evaluated by live/dead staining. In detail, chondrocytes were seeded into 6-well plates with a density of 5 × 10^5^ cells per well. After 24 h of culture, the chondrocytes were attached and the medium was removed. Subsequently, chondrocytes were incubated with different concentrations of CuMn-ZIF for 24 h. The wells were washed with PBS buffer twice. The chondrocytes were stained with calcein-AM/propidium iodide (PI) (Beyotime, China) for 5 min. Finally, the chondrocytes were imaged by a fluorescent microscope (Echo Revolve, China) and quantified by ImageJ software.

Cell viability was determined using a fixable viability dye, Zombie NIR (BioLegend, catalog no. 423106), which labels dead cells by covalently binding to amine groups on compromised cell membranes. Briefly, cells were harvested and washed twice with cold PBS. Subsequently, cells were resuspended in 100 μl of PBS and stained with Zombie NIR dye at a dilution of 1:1,000 (final concentration, 0.5 μg/ml) for 20 min at room temperature (25 °C) in the dark. After incubation, cells were washed twice with cold fluorescence-activated cell sorting (FACS) buffer (PBS containing 2% FBS and 0.1% sodium azide) to remove excess dye. Stained cells were analyzed using a BD flow cytometer. Viable cells were defined as Zombie NIR-negative, while dead/damaged cells were Zombie NIR-positive.

### Hemolysis assay

Fresh human venous blood was anticoagulated with heparin sodium (10 U/ml) or EDTA-K2 (1.5 mg/ml). Packed red blood cells (RBCs) were isolated by centrifugation at 1,500*g* for 10 min at room temperature. The RBC pellet was subsequently washed 3 times with sterile PBS (pH 7.4), followed by preparation of a 2% (v/v) RBC suspension in physiological saline. For hemolysis testing, 0.2 ml of the RBC suspension was combined with 1.8 ml of test solution in sterile tubes (final volume, 2 ml). Following incubation under specified conditions, the reaction mixtures were centrifuged at 1,500*g* for 10 min to pellet intact erythrocytes. Aliquots (200 μl) of the supernatant were carefully transferred to a 96-well microplate, and hemoglobin release was quantified by measuring absorbance at 540 nm using a microplate reader.

### ROS level detection

The dichlorodihydrofluorescein diacetate (DCFH-DA) probe (1:1,000 dilution in serum-free medium) was applied to 6-well plates (1 ml per well) and incubated (37 °C, 20 min, 5% CO₂). After incubation, cells underwent 3 serum-free medium washes prior to FACS.

### Mitochondrial membrane potential detection

The old culture medium was replaced with the 12-well plate and was washed once with PBS buffer. Subsequently, JC-1 working solution (1 ml) was applied to cells with gentle mixing, followed by incubation (5% CO₂, 37 °C, 20 min). After staining, the supernatant was removed and the cells were washed several times with JC-1 buffer. After adding complete medium (2 ml), mitochondrial membrane potential was assessed by confocal microscopy.

### Safety evaluation

The staining of major organs with hematoxylin and eosin (H&E), including the lung, liver, spleen, and heart, detected no tissue damage in either the PBS group or the other treatment groups. Tissue samples measuring less than 1 cm × 1 cm were used for the analysis. To prepare the tissues for examination, they were dehydrated with formalin buffer and varying concentrations of xylene and ethanol. The dehydrated tissues were then stained with H&E to assess any changes relative to the control tissues.

The serum levels of alanine transaminase (ALT), aspartate aminotransferase (AST), blood urea nitrogen (BUN), and creatinine (CRE) were measured by standard kits.

### Statistical methods

All statistical analyses are presented in the form of means ± standard deviation (SD). For result analysis, we employed GraphPad Prism software. Multiple groups meeting the assumption of normality were analyzed using one-way analysis of variance (ANOVA), followed by Tukey’s post hoc test. For comparing differences between 2 groups, Student’s *t* test was utilized. Statistical significance was defined as a *P* value of <0.05.

## Results

### Synthesis and structural elucidation of CuMn-ZIF nanozyme

**Fig. 2.**
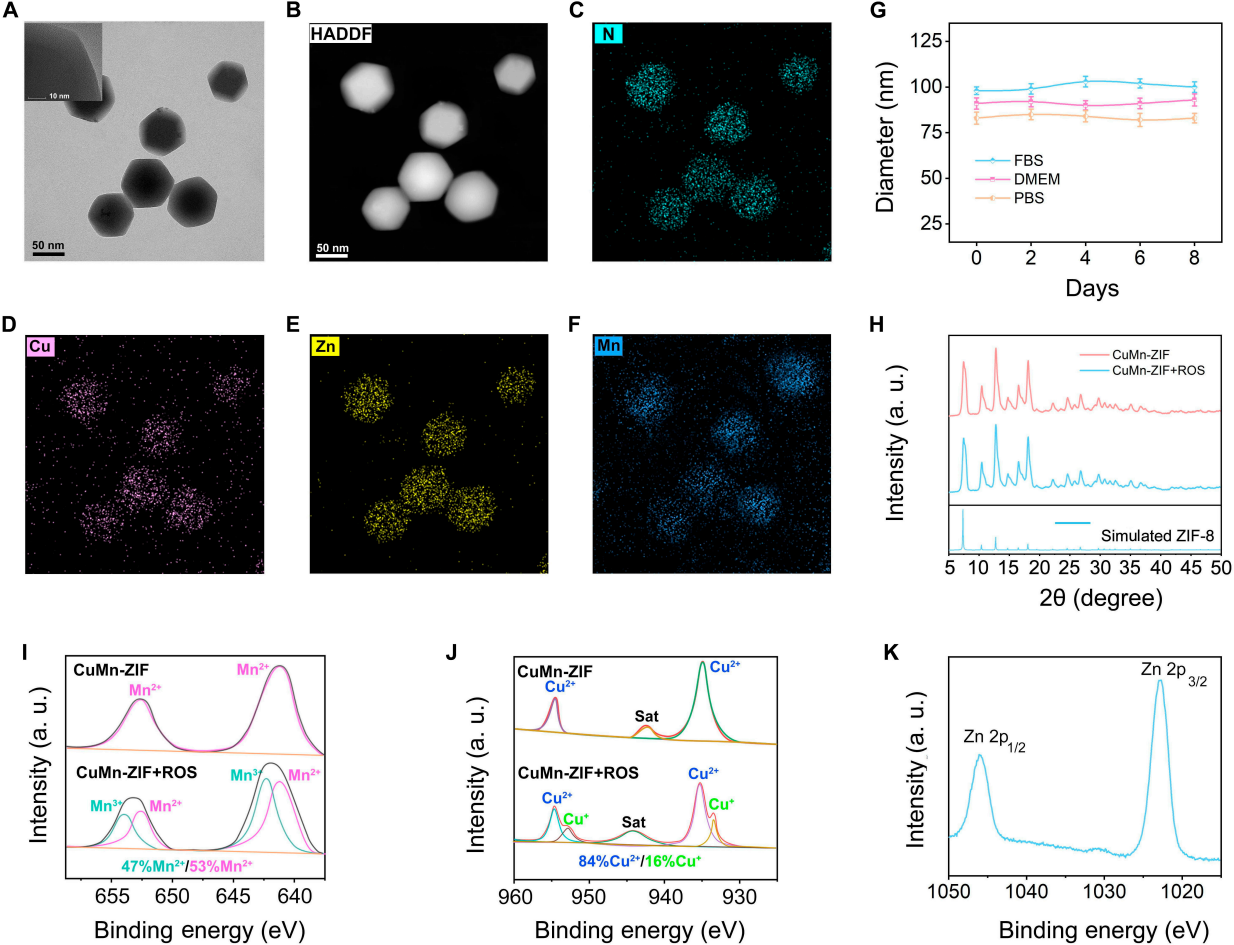
Synthesis and characterization of CuMn-ZIF nanozyme. (A) Representative transmission electron microscopy (TEM) images, high-resolution imaging, and corresponding high-angle annular dark-field (HAADF) images of CuMn-ZIF nanozyme. (B) Elemental mapping images for (C) N, (D) Cu, (E) Zn, and (F) Mn elements within the CuMn-ZIF nanozyme. (G) Size variation of CuMn-ZIF nanozyme monitored by dynamic light scattering (DLS) in fetal bovine serum (FBS), Dulbecco’s modified Eagle’s medium (DMEM), and phosphate-buffered saline (PBS) over 8 d. (H) X-ray diffraction (XRD) patterns of CuMn-ZIF nanozyme with and without ROS treatment, compared with the standard ZIF-8 reference. Representative high-resolution XPS analyses of (I) Mn, (J) Cu with and without ROS treatment, and (K) Zn in CuMn-ZIF.

A CuMn-ZIF bimetallic catalyst was synthesized at room temperature by incorporating Cu^2+^ into a metal–organic framework (MOF) that had been self-assembled from Zn^2+^ and 2-methylimidazole in methanol solution. The solution color changed from colorless to bule during the reaction, indicating the coordination of Cu^2+^ ions with the Zn-MOF. Transmission electron microscopy (TEM) and high-resolution image (Fig. 2A), high-angle annular dark-field imaging (HAADF) analysis (Fig. 2B), and mapping images (Fig. 2C to F) were used to analyze the morphology and elemental mapping of the CuMn-ZIF nanozyme. The analyses unveiled the presence of Cu, Zn, and N elements, along with their uniform distribution and a consistent size of approximately 74 nm. The nanoparticles exhibited excellent stability in PBS, cell culture medium [Dulbecco’s modified Eagle’s medium (DMEM)], and FBS for over 1 week, with hydrodynamic sizes of approximately 91.4 nm in PBS, 93.4 nm in DMEM, and 100.4 nm in FBS (Fig. 2G). The corresponding photos shown in Fig. S1 demonstrate that the designed CuMn-ZIF nanozyme maintained its integrity and uniform dispersion throughout the entire observation period (0 to 8 d), highlighting its robust performance in biologically relevant conditions.

The x-ray diffraction (XRD) patterns depicted in Fig. 2H for CuMn-ZIF subjected to conditions with and without ROS, as well as for Zn-MOF (ZIF), align closely with the characteristic pattern of ZIF-8. This alignment suggests that the crystalline framework of the material remains largely unperturbed by ROS exposure. The close alignment also confirms the high phase purity of both materials and demonstrates that the introduction of Cu and Mn has minimal influence on the structural integrity of the ZIF-8 (Zn-MOF) framework. Furthermore, the TEM image of CuMn-ZIF following ROS treatment is shown in Fig. S2; the regular hexagonal shape further substantiates the resilience and structural stability of CuMn-ZIF nanozyme.

The zeta potential of ZIF increases from +8 mV to +12 mV upon Cu^2+^ doping (Fig. S3A), thereby confirming the successful incorporation of Cu^2+^ ions. Furthermore, the Fourier transform infrared (FTIR) (Fig. S3B) and Raman spectra (Fig. S3C) yield congruent results, revealing that the spectral profile of CuMn-ZIF closely aligns with that of Zn-MOF. The x-ray photoelectron spectroscopy (XPS) analysis (Fig. 2I to K) revealed the characteristic peaks of Mn (641/653 eV), Cu (934/955 eV), and Zn (1,022/1,046 eV), verifying the successful synthesis of CuMn-ZIF nanozyme. It reveals that Mn in CuMn-ZIF transitions from a predominantly Mn^2+^ state to a mixed Mn^2+^/Mn^3+^ state, with the Mn^3+^ fraction increasing to 47% after ROS treatment. This shift highlights Mn’s critical role in ROS scavenging via redox mechanisms. Concurrently, Cu shifts from a predominantly Cu^2+^ state to a coexistence of Cu^+^ and Cu^2+^, with the Cu^+^ fraction rising to 16%. This indicates Cu’s active catalytic role in ROS elimination. These findings underscore the dynamic redox capabilities of CuMn-ZIF, which are essential for its catalytic efficacy.

### ROS elimination ability of CuMn-ZIF nanozyme in vitro

**Fig. 3.**
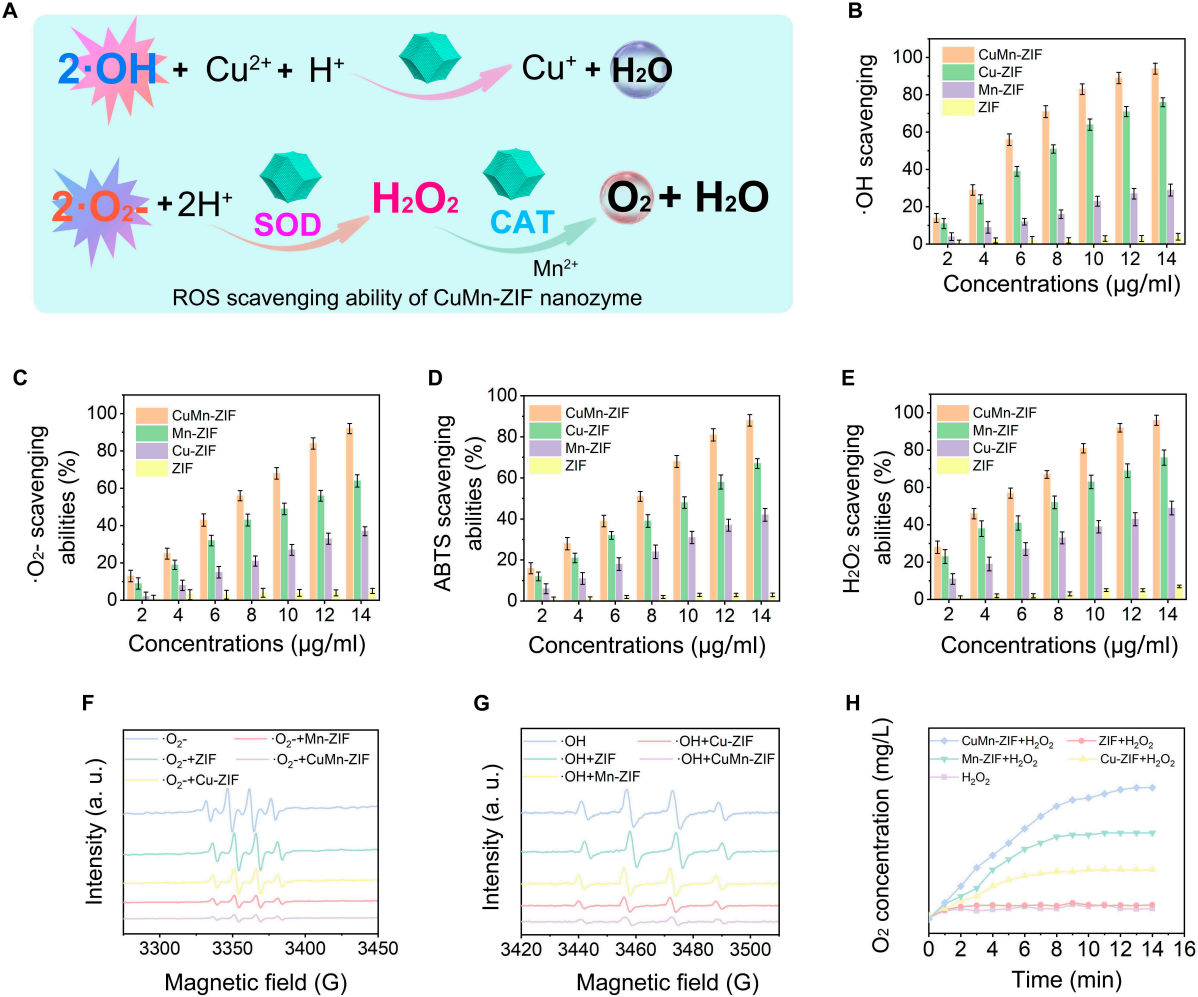
In vitro dual-enzyme ROS scavenging of CuMn-ZIF nanozyme. (A) Schematic representation of the ROS depletion ability of CuMn-ZIF nanozyme. Elimination of (B) •OH, (C) •O_2_^−^, (D) ABTS, and (E) H_2_O_2_ treated with CuMn-ZIF, Cu-ZIF, Mn-ZIF, and ZIF nanozyme, respectively. Electron spin resonance (ESR) spectra of (F) •O_2_^−^, (G) •OH, and (H) the H_2_O_2_ decomposition and O_2_ generation treated with CuMn-ZIF, Cu-ZIF, Mn-ZIF, and ZIF nanozyme, respectively.

The accompanying schematic provides a concise depiction of the CuMn-ZIF nanozyme’s ROS scavenging mechanism in Fig. 3A, which is driven by 2 primary catalytic pathways. Initially, Cu^2+^ ions within the CuMn-ZIF framework mediate the reduction of •OH to Cu^+^ and H_2_O. Subsequently, Mn^2+^ initiates the conversion of •O_2_^−^ to H_2_O_2_ while being oxidized to Mn^3+^. Subsequently, H_2_O_2_ is catalytically decomposed into O_2_ and H_2_O by Mn^2+^. These reactions effectively replicate the enzymatic functions of SOD and CAT of CuMn-ZIF nanozyme. Specifically, •OH gained electrons in the presence of H^+^ and was converted to H_2_O. •O_2_^−^ was first converted to H_2_O_2_ through the SOD-mimic activity of CuMn-ZIF nanozyme, after H_2_O_2_ was decomposed to O_2_ and H_2_O via the CAT-mimic activity of the nanozyme.

CuMn-ZIF nanozyme displayed concentration-dependent ROS scavenging capacity, eliminating ~94% of •OH (Fig. 3B), 92% of •O_2_^−^ (Fig. 3C), 88% of ABTS (Fig. 3D), and 96% of H_2_O_2_ (Fig. 3E) at 14 μg/ml—significantly outperforming monometallic counterparts (Cu-ZIF, Mn-ZIF) and pure ZIF.

Electron spin resonance (ESR) spectroscopy was employed to validate these enzymatic activities. Figure 3F presents the ESR spectra of •O_2_^−^ treated with various nanozymes: CuMn-ZIF exhibited the most significant signal reduction, substantially outperforming Cu-ZIF and Mn-ZIF, which showed moderate activity, while ZIF alone had negligible effect. This confirms the superior SOD-mimetic activity of the bimetallic nanozyme. Similarly, Fig. 3G illustrates the •OH ESR spectra, where CuMn-ZIF demonstrated the strongest signal attenuation compared to its monometallic counterparts, highlighting its enhanced antioxidant activity. These results substantiate the dual enzymatic capabilities of CuMn-ZIF in neutralizing both •O_2_^−^ and •OH.

To investigate the capacity of CuMn-ZIF nanozymes to generate H_2_O_2_, we incorporated the nanozymes into a reaction milieu that included •O_2_^−^, which was produced by the xanthine/xanthine oxidase system. Luminol, a chemiluminescent indicator, was employed to detect the presence of H_2_O_2_. The chemiluminescence was quantified using a sensitive imaging system. As illustrated in Fig. S4, the experimental outcomes demonstrate a marked escalation in chemiluminescent signal intensity concomitant with the consumption of •O_2_^−^.

The CAT-mimic activity was quantified by monitoring O_2_ generation upon incubation with H_2_O_2_. While ZIF alone showed minimal O_2_ production, both Cu-ZIF and Mn-ZIF exhibited moderate activity. In contrast, CuMn-ZIF with H_2_O_2_ generated O_2_ efficiently, increasing from 7.2 mg/ml to 13.5 mg/ml within 14 min (Fig. 3H). This pronounced enhancement over monometallic controls evidences clear synergistic effects in the bimetallic system, with bubble formation visually confirming O_2_ production (Fig. S5).

### In vitro effect of CuMn-ZIF nanozyme on metabolites

**Fig. 4.**
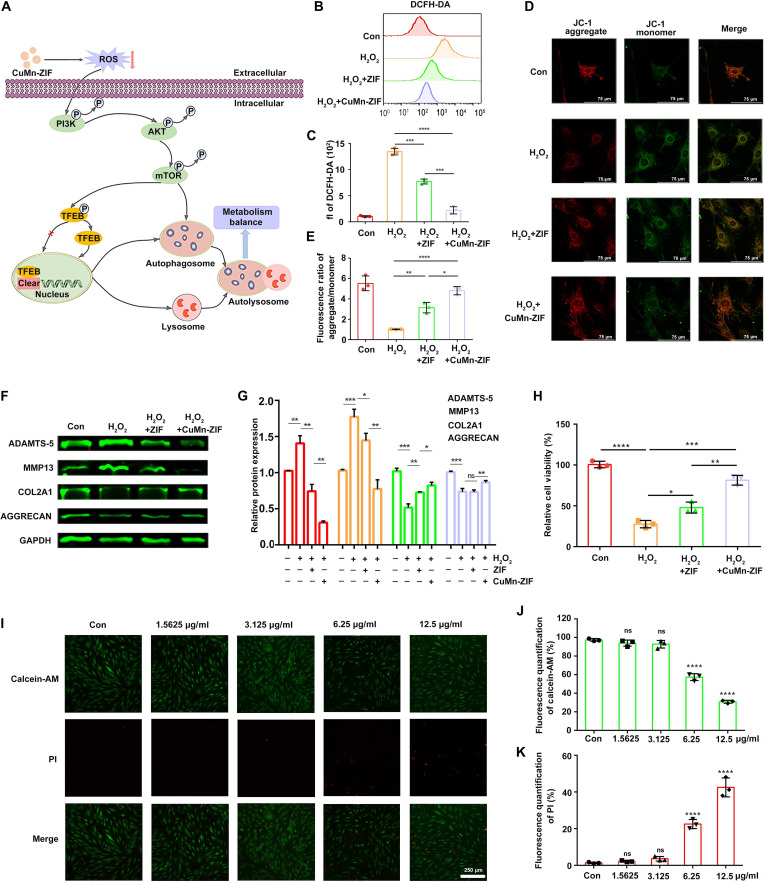
CuMn-ZIF nanozymes mitigate oxidative stress and protect chondrocytes in vitro. (A) Schematic diagram illustrating the proposed mechanism of CuMn-ZIF nanozymes in treating OA. CuMn-ZIF scavenges ROS, inhibits the phosphorylation of the PI3K–AKT–mTOR pathway, promotes TFEB nuclear translocation, enhances autophagic flux, and ultimately restores the balance between anabolism and catabolism in chondrocytes. (B) Intracellular ROS levels were measured using the DCFH-DA probe and (C) analyzed by flow cytometry following treatment with PBS, H_2_O_2_, H_2_O_2_ + ZIF, or H_2_O_2_ + CuMn-ZIF. (D) Representative fluorescence images and (E) quantitative analysis of mitochondrial membrane potential assessed by JC-1 staining (scale bar, 75 μm). Red fluorescence indicates JC-1 aggregates (healthy mitochondria), and green fluorescence indicates JC-1 monomers (depolarized mitochondria). (F) Representative WB images and (G) corresponding quantitative analysis of the protein expression levels of catabolic markers (ADAMTS-5, MMP-13) and anabolic markers (COL2A1, AGGRECAN). (H) Cell viability was determined by CCK-8 assay after treatment with PBS, H_2_O_2_, H_2_O_2_ + ZIF, or H_2_O_2_ + CuMn-ZIF. (I) Live/dead cell staining images (scale bar, 250 μm) using calcein-AM (green, live cells) and PI (red, dead cells), and the corresponding quantitative analysis of (J) calcein-AM and (K) PI fluorescence intensity. Statistical analysis was conducted using one-way ANOVA analysis. Data were represented as mean ± SD (*n* = 3). ****P < 0.0001, ***P < 0.001, **P < 0.01, *P < 0.05, ns: not significant.

CuMn-ZIF nanozyme attenuates OA by scavenging ROS and restoring autophagic flux. Mechanistically, CuMn-ZIF suppresses ROS-induced activation of the PI3K–AKT–mTOR pathway, leading to TFEB dephosphorylation and nuclear translocation. This cascade enhances autophagosome–lysosome fusion, ultimately rebalancing cartilage metabolism by up-regulating anabolic factors and down-regulating catabolic enzymes, thereby mitigating chondrocyte injury (Fig. 4A).

To evaluate the antioxidant capacity of the CuMn-ZIF nanozyme, intracellular ROS levels were measured using the DCFH-DA fluorescent probe and analyzed by flow cytometry. While ZIF partially reduced ROS, CuMn-ZIF treatment exhibited a more pronounced reduction (Fig. 4B and C), highlighting its superior ROS scavenging capacity. Apoptosis was monitored via JC-1 staining to evaluate mitochondrial membrane potential [24]. H_2_O_2_ treatment enhanced green fluorescence, indicating apoptosis activation, whereas CuMn-ZIF treatment notably reversed this effect, restoring red fluorescence and demonstrating a potent anti-apoptotic effect (Fig. 4D and E).

We further investigated the influence of CuMn-ZIF on cartilage homeostasis by analyzing the expression of key metabolic markers in H_2_O_2_-stimulated primary OA chondrocytes. Western blot (WB) analysis showed that CuMn-ZIF significantly suppressed catabolic proteins (ADAMTS-5 and MMP-13) and enhanced anabolic proteins (COL2A1 and AGGRECAN) compared to other treatment groups (Fig. 4F and G). These results suggest that CuMn-ZIF exerts a protective effect against cartilage degradation by rebalancing metabolic homeostasis, underscoring its potential as a therapeutic agent for mitigating OA progression. Cell viability assessed by CCK-8 assay showed that CuMn-ZIF nanozyme significantly promoted chondrocyte viability (Fig. 4H), suggesting that its beneficial effect on metabolic balance translates into functional cellular recovery.

Biocompatibility assessments further supported its potential therapeutic utility. CCK-8 assays further confirmed that chondrocyte viability remained uncompromised at 1.5625 to 3.125 μg/ml, although a gradual decrease was observed at ≥6.25 μg/ml (Fig. S6). Both calcein-AM/PI staining (Fig. 4I to K) and Zombie NIR (Fig. S7) demonstrated high cell viability and minimal cytotoxicity at concentrations up to 3.125 μg/ml. Collectively, these results establish the favorable biocompatibility of CuMn-ZIF nanozymes. Moreover, hemocompatibility was evaluated by measuring hemoglobin release. Even at 400 μg/ml, CuMn-ZIF induced less than 5% hemolysis (Fig. S8), demonstrating excellent blood compatibility and reinforcing its potential for clinical application.

### CuMn-ZIF nanozyme enhances autophagic flux in vitro

**Fig. 5.**
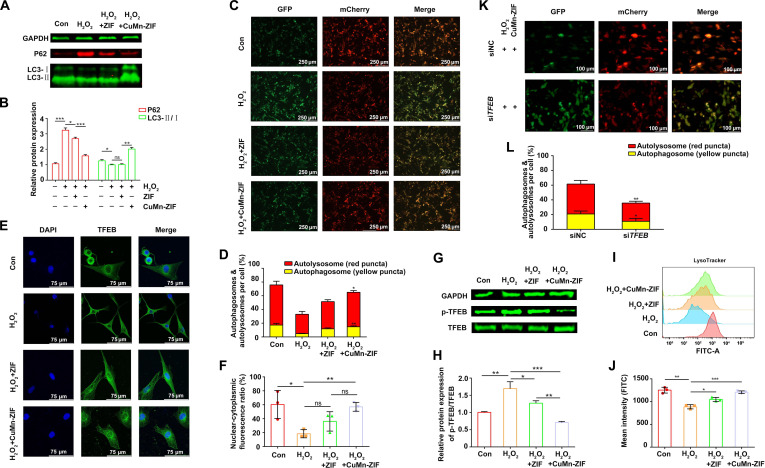
CuMn-ZIF nanozymes enhance autophagic flux and promote TFEB nuclear translocation in chondrocytes in vitro. (A) Representative WB images and (B) quantitative analysis of autophagy-related proteins (LC3-II/I and P62) in chondrocytes treated with PBS, H_2_O_2_, H_2_O_2_ + ZIF, or H_2_O_2_ + CuMn-ZIF. (C) Representative confocal images and (D) quantitative analysis of autophagic flux using the mCherry-GFP-LC3 fluorescent probe. Yellow puncta (mCherry^+^GFP^+^) represent autophagosomes, and red puncta (mCherry^+^GFP^−^) represent autolysosomes (scale bar, 250 μm). (E) Immunofluorescence images showing the subcellular localization of TFEB (FITC, green) with nuclei counterstained by DAPI (blue), and (F) the corresponding statistical analysis of nuclear-to-cytoplasmic TFEB fluorescence intensity ratio (scale bar, 75 μm). (G) Representative WB images of p-TFEB and total TFEB and (H) quantitative analysis of p-TFEB/TFEB protein levels. (I) Flow cytometry analysis and (J) quantification of lysosomal pH using LysoTracker Red staining. (K) Representative confocal images and (L) quantitative analysis of autophagic flux using the mCherry-GFP-LC3 fluorescent probe according to siNC and siTFEB treatment (scale bar, 100 μm). Statistical analysis was conducted using one-way ANOVA analysis. Data were represented as mean ± SD (*n* = 3). ****P < 0.0001, ***P < 0.001, **P < 0.01, *P < 0.05, ns: not significant.

Having established the protective effect of CuMn-ZIF on cartilage metabolism, we next investigated whether its mechanism involves the regulation of autophagy, a self-degradative process essential for cellular homeostasis and whose dysfunction is implicated in OA pathogenesis [25–27]. WB analysis revealed that H_2_O_2_ treatment led to the accumulation of P62, indicating impaired autophagic degradation, whereas CuMn-ZIF exhibited excellent capacity to reduce P62 levels. Consistent with this, the LC3-II/LC3-I ratio, a key indicator of autophagosome formation, was significantly elevated in CuMn-ZIF-treated cells (Fig. 5A and B). These results demonstrate that CuMn-ZIF effectively restores autophagic activity in OA chondrocytes.

However, the initiation of autophagy does not guarantee successful outcomes, as its efficacy depends on the smooth progression of autophagic flux [28,29]. mCherry-GFP-LC3 adenovirus infection in cells is an important method to monitor autophagic flux [30,31]. We observed that CuMn-ZIF promoted autophagosome–lysosome fusion, as shown by increased red puncta and GFP quenching (Fig. 5C and D), indicating restored autophagic flux in OA chondrocytes.

Next, we investigated whether CuMn-ZIF modulates lysosomal function, which is essential for autophagic completion. Given that TFEB—a master transcription factor governing lysosomal biogenesis and autophagy—is negatively regulated by mTORC1 [32], we examined its subcellular localization. Immunofluorescence imaging revealed that H_2_O_2_ treatment retained TFEB in the cytoplasm, whereas CuMn-ZIF facilitated its nuclear translocation (Fig. 5E and F). WB analysis showed that the H_2_O_2_ group had the highest TFEB phosphorylation level, and the CuMn-ZIF group perfectly reversed this phenomenon (Fig. 5G and H). We further assessed lysosomal activity using LysoTracker staining and flow cytometry. The results indicated that CuMn-ZIF exhibited a strong acidity compared to other groups (Fig. 5I and J). To confirm the role of TFEB in the autophagy process, TFEB was knocked down in chondrocytes using siRNA. As shown in Fig. 5K and L, TFEB knockdown significantly weakened red fluorescence and reduced GFP quenching. Taken together, these results demonstrate that CuMn-ZIF enhances autophagic flux by promoting TFEB-mediated autophagosome–lysosome fusion in OA chondrocytes.

### Inhibition of the PI3K–AKT–mTOR pathway by CuMn-ZIF nanozyme in vitro

**Fig. 6.**
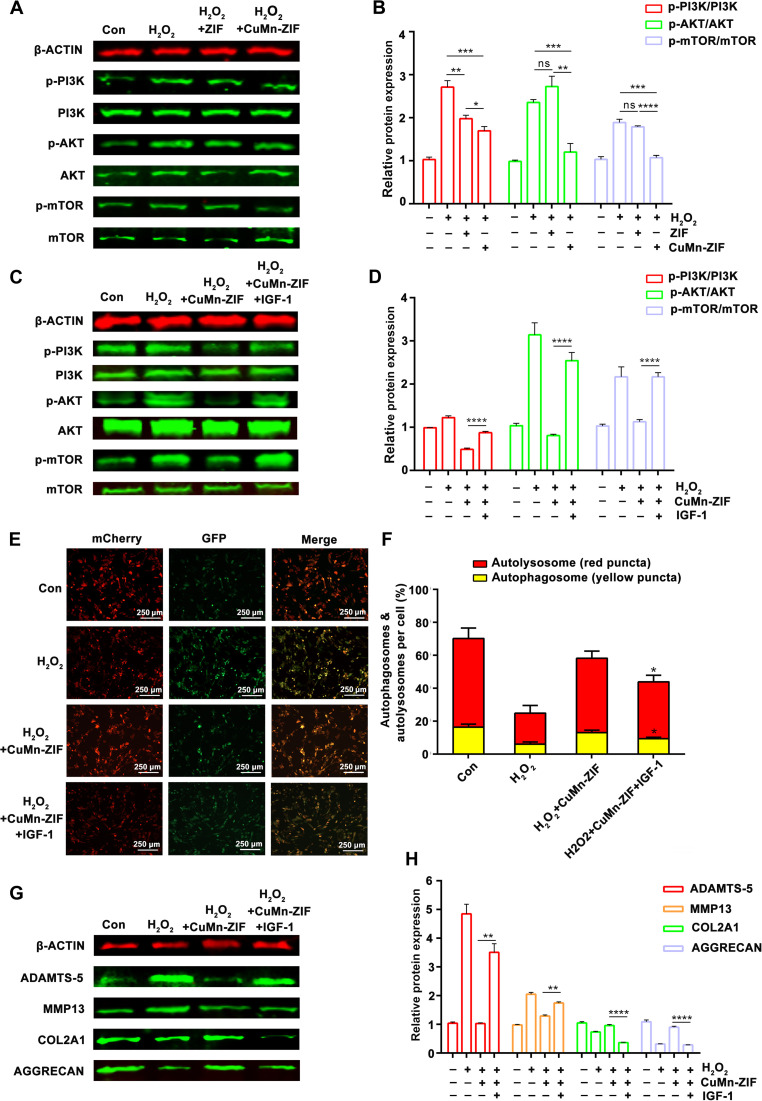
CuMn-ZIF nanozymes exert protective effects by inhibiting the PI3K–AKT–mTOR pathway in vitro. (A) Representative WB images and (B) quantitative analysis of key proteins in the PI3K–AKT–mTOR pathway (p-PI3K/PI3K, p-AKT/AKT, p-mTOR/mTOR) in chondrocytes treated with BS, H_2_O_2_, H_2_O_2_ + ZIF, or H_2_O_2_ + CuMn-ZIF. (C) WB images and (D) quantitative analysis of WB analysis of p-PI3K/PI3K, p-AKT/AKT, and p-mTOR/mTOR expression in chondrocytes treated with PBS, H_2_O_2_, H_2_O_2_ + CuMn-ZIF, and H_2_O_2_ + CuMn-ZIF + IGF-1 (PI3K agonist). (E) Representative confocal images and (F) quantitative analysis of autophagic flux using the mCherry-GFP-LC3 fluorescent probe treated with PBS, H_2_O_2_, H_2_O_2_ + CuMn-ZIF, and H_2_O_2_ + CuMn-ZIF + IGF-1. Yellow puncta (autophagosome) and red puncta (autolysosomes) (scale bar, 250 μm). (G) Representative WB images and (H) quantitative analysis of ADAMTS-5, MMP-13, COL2A1, and AGGRECAN protein expression in chondrocytes. Statistical analysis was conducted using one-way ANOVA analysis. Data were represented as mean ± SD (*n* = 3). ****P < 0.0001, ***P < 0.001, **P < 0.01, *P < 0.05, ns: not significant.

The PI3K–AKT–mTOR signaling pathway plays a central role in regulating autophagy and has been strongly implicated in OA pathogenesis [33–35]. To determine whether CuMn-ZIF influences this pathway, we assessed the phosphorylation levels of PI3K, AKT, and mTOR. WB analysis revealed that H_2_O_2_ treatment markedly enhanced the phosphorylation of all 3 proteins, whereas CuMn-ZIF significantly inhibited their activation (Fig. 6A and B).

To establish whether CuMn-ZIF acts directly through the PI3K–AKT–mTOR axis, we employed the PI3K-specific agonist insulin-like growth factor 1 (IGF-1) in a rescue experimental setting. As shown in Fig. 6C and D, IGF-1 cotreatment effectively reversed the CuMn-ZIF-induced reduction in phosphorylation of PI3K, AKT, and mTOR. We further evaluated the functional impact of this reversal on autophagy using the mCherry-GFP-LC3 reporter. IGF-1 treatment reduced red puncta and impaired autolysosome formation, indicating that reactivation of PI3K signaling abrogates CuMn-ZIF-mediated restoration of autophagic flux (Fig. 6E and F).

We further assessed whether IGF-1 could counteract the therapeutic benefits of CuMn-ZIF on chondrocyte metabolic homeostasis. WB analysis revealed that cotreatment with IGF-1 reversed the protective effects of CuMn-ZIF, resulting in decreased expression of COL2A1 and Aggrecan, along with increased expression of ADAMTS-5 and MMP-13 (Fig. 6G and H). Collectively, these findings demonstrate that CuMn-ZIF mitigates ROS-induced chondrocyte injury and metabolic imbalance primarily through suppression of the PI3K–AKT–mTOR signaling pathway.

### CuMn-ZIF nanozyme ameliorates OA progression in vivo

**Fig. 7.**
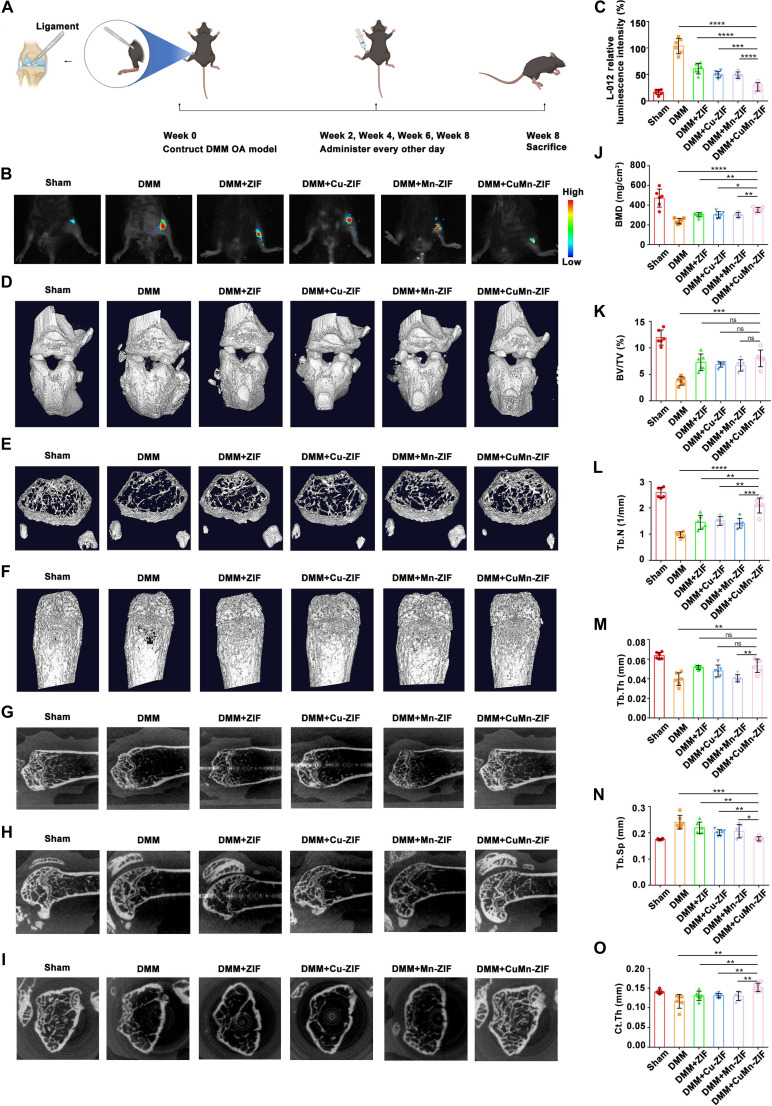
CuMn-ZIF nanozymes alleviate OA progression in vivo. (A) Schematic flowchart of the in vivo experimental design. Mice were randomly divided into 6 groups (*n* = 6 per group): Sham, DMM, DMM + ZIF, DMM + Cu-ZIF, DMM + Mn-ZIF, and DMM + CuMn-ZIF. Nanozymes were intra-articularly injected every other day at weeks 2, 4, 6, and 8 post-surgery. (B) In vivo imaging of ROS levels in the knee joints using the L-012 chemiluminescent probe and (C) quantitative analysis of the luminescence intensity. (D) Representative 3D micro-CT reconstruction images of mouse knee joints. (E to I) Representative 2D micro-CT images in the coronal, sagittal, and horizontal planes, highlighting subchondral bone changes. (J to O) Quantitative analysis of micro-CT parameters: bone mineral density (BMD), bone volume/total volume (BV/TV), trabecular number (Tb.N), trabecular thickness (Tb.Th), trabecular separation (Tb.Sp), and cortical thickness (Ct.Th). Data are presented as the mean ± SD (*n* = 6 mice per group). *P* values were calculated using one-way ANOVA with Tukey’s post hoc test. ****P < 0.0001, ***P < 0.001, **P < 0.01, or *P < 0.05, ns: not significant.

The in vivo therapeutic efficacy of CuMn-ZIF in treating OA was further investigated in the mouse model, which was induced using a widely employed DMM model (Fig. 7A). The chemiluminescent probe L-012 was used to monitor the intra-articular ROS levels. Luminescence imaging and quantitative analysis revealed a substantial increase in ROS in the DMM group, which was moderately reduced by treatment with ZIF, Cu-ZIF, or Mn-ZIF. In contrast, CuMn-ZIF treatment led to a significantly more pronounced reduction in ROS signals (Fig. 7B and C), underscoring its superior ROS scavenging capacity in vivo.

Micro-CT analysis demonstrated severe joint space narrowing, osteophyte formation, and architectural disruption in DMM mice relative to Sham controls (Fig. 7D). Three-dimensional (3D) reconstruction and multi-planar 2D views further revealed extensive bone deterioration in the DMM group (Fig. 7E to I). While monometallic ZIF, Cu-ZIF, and Mn-ZIF treatments conferred modest structural protection, CuMn-ZIF administration most effectively preserved joint morphology, inhibited osteophyte development, and restored bone microarchitecture. Quantitative analysis of bone parameters confirmed that CuMn-ZIF significantly improved BMD (1.5×), trabecular bone volume fraction (BV/TV, 2.1×), trabecular number (Tb.N, 2×), and cortical thickness (Ct.Th, 1.4×) while reducing trabecular spacing (Tb.Sp, 0.77×) compared to other groups (Fig. 7J to O).

**Fig. 8.**
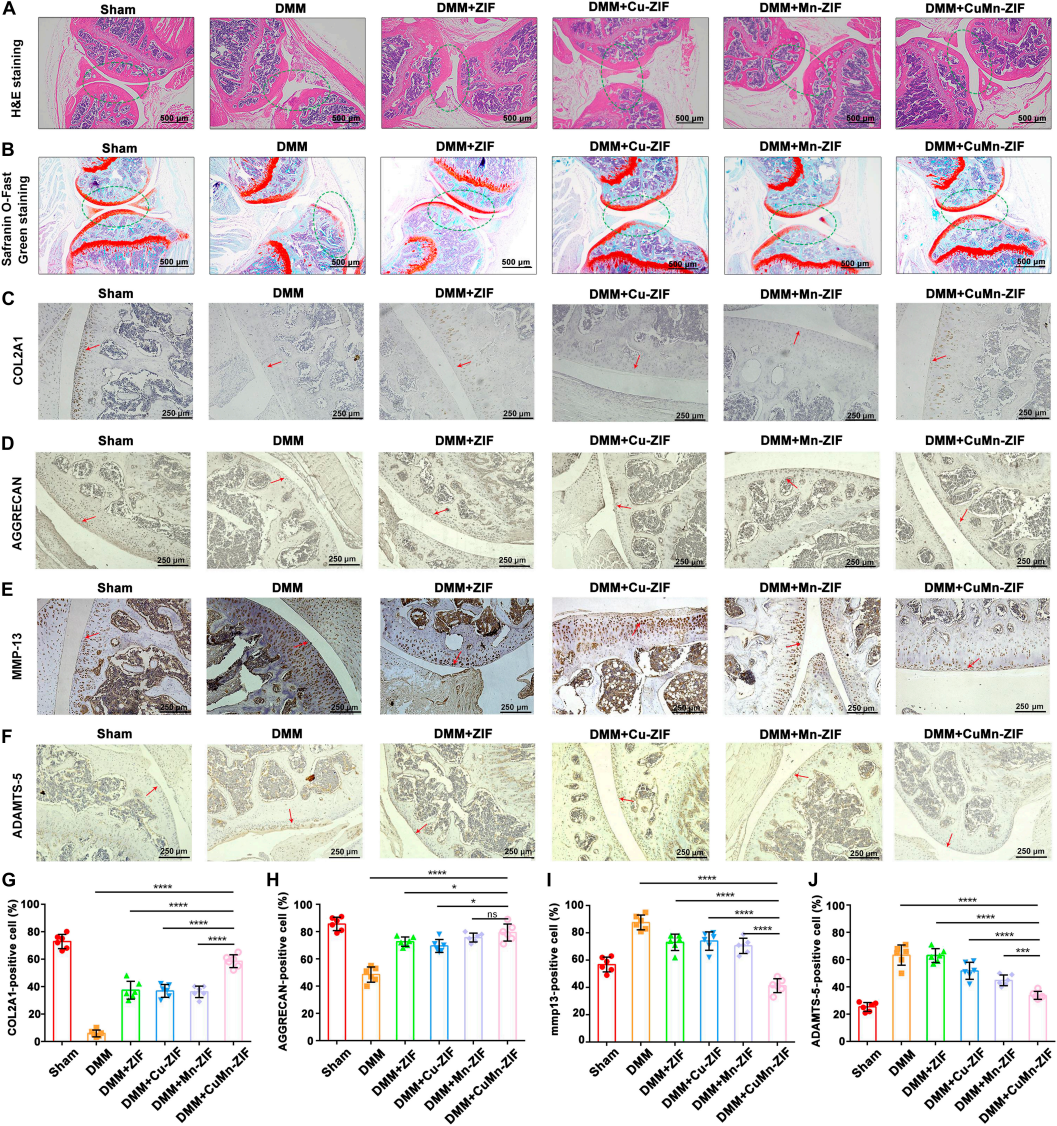
CuMn-ZIF nanozymes improve histological outcomes and matrix metabolism in vivo. (A) Representative H&E staining images and (B) Safranin-O/Fast Green staining images of the OA articular tissues (scale bar, 500 μm). (C to F) Representative immunohistochemical staining images for (C) COL2A1, (D) AGGRECAN, (E) MMP-13, and (F) ADAMTS-5 in joint tissues (scale bar, 250 μm). Red arrows indicate positive staining. (G to J) Quantitative analysis of the integrated optical density (IOD) for (G) COL2A1, (H) AGGRECAN, (I) MMP-13, and (J) ADAMTS-5, performed using ImageJ software. Data are expressed as the mean ± SEM (*n* = 6). ****P < 0.0001, ***P < 0.001, **P < 0.01, or *P < 0.05. Data are presented as the mean ± SD (*n* = 6 mice per group). *P* values were calculated using one-way ANOVA with Tukey’s post hoc test. ****P < 0.0001, ***P < 0.001, **P < 0.01, or *P < 0.05, ns: not significant.

At the histological level, mouse joints were initially subjected to Osteoarthritis Research Society International (OARSI) scoring (Fig. S9). Histopathological evaluation using H&E and Safranin-O/Fast Green staining (Fig. 8A and B) indicated severe cartilage erosion, proteoglycan loss, and subchondral bone damage in DMM mice. All nanozyme treatments attenuated these degenerative changes, with CuMn-ZIF exhibiting the most substantial cartilage preservation and structural maintenance. Immunohistochemical analysis showed that CuMn-ZIF robustly enhanced the expression of COL2A1 and AGGRECAN (Fig. 8C, D, G, and H) and suppressed the expression of MMP-13 and ADAMTS-5 (Fig. 8E, F, I, and J), outperforming both ZIF and the single-metal nanozymes.

**Fig. 9.**
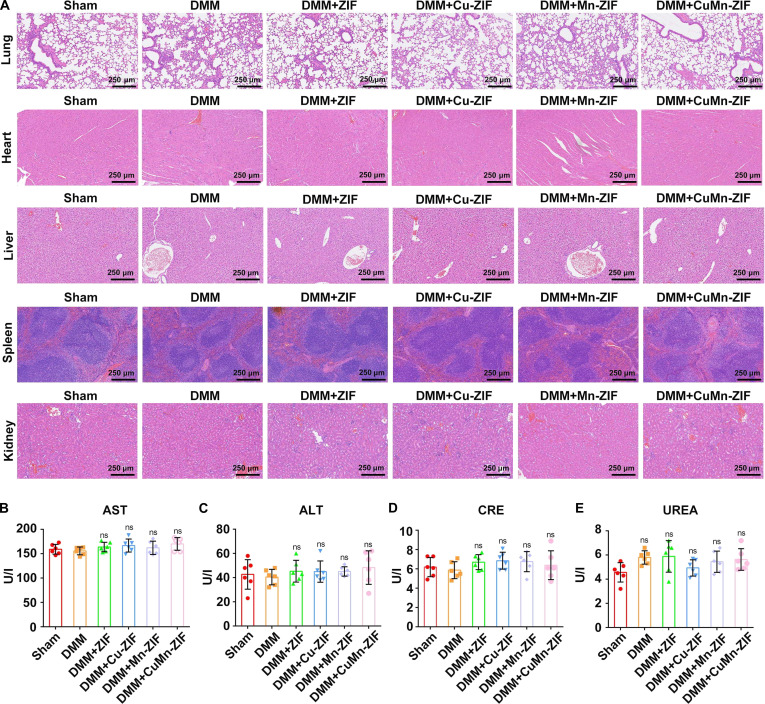
Biosafety evaluation of CuMn-ZIF nanozyme in vivo. (A) Hematology data of C57/BL6 mice treated with Sham, DMM, DMM + ZIF, DMM + Cu-ZIF, DMM + Mn-ZIF, and DMM + CuMn-ZIF. Histology staining images of major organs of mice with different treatments were obtained. The scale bar for these images is 250 μm. Serum biomarkers were analyzed to assess organ toxicity: AST (B) and ALT (C) for hepatotoxicity; BUN (D) and CRE (E) for nephrotoxicity. Data are presented as the mean ± SD (*n* = 6 mice per group). *P* values were calculated using one-way ANOVA with Tukey’s post hoc test. ****P < 0.0001, ***P < 0.001, **P < 0.01, or *P < 0.05, ns: not significant.

Biosafety evaluation of CuMn-ZIF nanozyme showed no evident tissue damage in major organs (lung, liver, spleen, heart) in H&E-stained sections from all groups (Fig. 9A). The representative blood biochemical indicators of ALT, aspartate AST, BUN, and CRE were measured after treatments. All these indexes were within the normal range, suggesting minimal hepatotoxicity and nephrotoxicity of the CuMn-ZIF nanozyme and supporting the biocompatibility of CuMn-ZIF nanozyme (Fig. 9B to E).

## Discussion

Nanozyme-based ROS scavenging offers a transformative approach to treating OA [36,37]. However, most current platforms focus solely on single enzymatic functions [38,39]. In this study, we developed a bimetallic CuMn-ZIF nanozyme that combines SOD and CAT mimetic activities within a single metal–organic framework, enabling synergistic ROS neutralization in the harsh oxidative microenvironment of OA joints. The nanozyme demonstrated strong catalytic performance, effectively eliminating ~94% of hydroxyl radicals and 92% of superoxide anions at low concentrations while also exhibiting exceptional stability and biocompatibility in vivo.

A key innovation is found in the synergistic Cu–Mn dual-metal center. While the monometallic Cu-ZIF and Mn-ZIF displayed modest activity, the bimetallic CuMn-ZIF exhibited significantly enhanced catalytic efficiency, converting H_2_O_2_ to O_2_ and H_2_O at rates that are twice as high as those of the individual components. This synergy reflects the function of natural bimetallic enzyme active sites and overcomes a critical limitation of conventional single-site nanozymes. The framework’s structural integrity remained intact after ROS exposure, with XPS revealing dynamic redox cycling—Mn^2+^/Mn^3+^ and Cu^+^/Cu^2+^ transitions—that underpins sustained catalytic activity. This mechanistic robustness distinguishes CuMn-ZIF from previously reported antioxidant nanomaterials that often suffer from activity loss under continuous oxidative stress.

Mechanistically, our results demonstrate that CuMn-ZIF alleviates OA progression by restoring ROS-impaired autophagic flux, a process known to be critically dysregulated in OA pathogenesis [40]. Accumulating evidence indicates that excessive ROS contributes to autophagosome–lysosome system dysfunction [41,42], yet few interventions successfully target this connection. In the present study, efficient ROS scavenging by CuMn-ZIF suppressed the PI3K–AKT–mTOR pathway, leading to TFEB dephosphorylation and nuclear translocation. This cascade enhanced lysosomal biogenesis and promoted autophagosome–lysosome fusion, as confirmed by elevated LC3-II/LC3-I ratios and efficient P62 degradation. Consequently, cartilage homeostasis was re-established, with significant down-regulation of catabolic enzymes and up-regulation of anabolic components. The pivotal role of TFEB in this process was validated through loss-of-function experiments, where TFEB knockdown substantially attenuated autophagic flux and compromised the therapeutic effects of CuMn-ZIF. Although TFEB activation has been proposed as a potential therapeutic strategy for OA, its functional connection with ROS modulation remains underexplored. Our study provides mechanistic evidence that CuMn-ZIF requires PI3K–AKT–mTOR inhibition to exert its chondroprotective effects, as validated by IGF-1 rescue assays. These findings define a coherent signaling pathway in OA treatment, linking ROS scavenging to the restoration of autophagy via the PI3K–AKT–mTOR–TFEB axis.

The therapeutic efficacy in the DMM model was substantial. A single intra-articular injection every 2 weeks led to an 80% reduction in intra-articular ROS. This treatment not only preserved cartilage integrity but also reversed subchondral bone loss, resulting in a 1.5-fold increase in BMD and a 2.1-fold increase in trabecular bone volume compared to untreated DMM mice. These effects markedly outperformed monometallic analogs and naive ZIF-8, underscoring the importance of dual-enzyme synergy. Notably, the treatment effect size is clinically relevant, approaching disease-modifying benefits rather than purely symptomatic ones.

From a translational perspective, CuMn-ZIF presents a promising approach for OA treatment, effectively addressing the limitations of NSAIDs by providing disease-modifying effects. Its stability in biological fluids (>8 d), favorable safety profile, and bimonthly dosing could enhance patient compliance. However, challenges persist, including the need to evaluate systemic distribution, long-term clearance, and biocompatibility in larger models. Furthermore, dose optimization, Good Manufacturing Practices (GMP) manufacturing, cost-effectiveness, and long-term safety require thorough assessment. Injection invasiveness and regulatory hurdles also need to be addressed. Combining CuMn-ZIF with emerging therapies or functional modifications may further enhance its efficacy. Overcoming these challenges is crucial for successful clinical translation. Beyond OA, the dual-enzyme design principles established here can be applied to other ROS-driven pathologies, including rheumatoid arthritis, neurodegenerative diseases, and ischemia–reperfusion injury. This work demonstrates that precise redox modulation can restore autophagic flux and cellular homeostasis, establishing a generalized blueprint for enzyme-mimetic nanomedicines.

Despite the promising results of this study, several limitations must be addressed. First, due to differences in species, age, and sex, none of the animal models can fully replicate all pathological processes of human OA. Invasive surgery facilitates a more uniform development of OA and is currently the most reliable animal model for this condition [43,44]. However, it is not suitable for studying the changes in OA that occur during long-term wear and aging. Future studies should include more convincing evidence to compare the efficiency of CuMn-ZIF nanozyme in different OA models. Second, while the PI3K/AKT/mTOR–autophagic flux axis is a key mechanism, additional pathways such as nuclear factor κB (NF-κB) may also contribute to CuMn-ZIF’s efficacy [5,45]. Future work should investigate these pathways to clarify the mode of action of the nanozyme. Third, while local modulation of oxidative stress and autophagy within the joint is crucial, emerging evidence suggests that systemic factors, such as the gut microbiota, play a critical role in OA progression. Notably, certain probiotics, particularly *Akkermansia muciniphila*, have been shown to reduce systemic inflammation and modulate immune responses, yielding beneficial effects on OA [46].

In summary, the CuMn-ZIF nanozyme platform offers a powerful, mechanism-based therapy for OA, effectively translating ROS scavenging into lasting chondroprotection. Its dual-enzyme synergy, strong mechanistic link to autophagy, and proven disease-modifying efficacy make it a promising candidate for clinical development.

## Ethical Approval

All animal experiments were approved by the Animal Experiment Ethics Committee of First Affiliated Hospital of Harbin Medical University (approval number: 2025002).

## Data Availability

Data will be made available on request.

## References

[1] Xue C, Tian J, Cui Z, Liu Y, Sun D, Xiong M, Yi N, Wang K, Li X, Wang Y, et al. Reactive oxygen species (ROS)-mediated M1 macrophage-dependent nanomedicine remodels inflammatory microenvironment for osteoarthritis recession. Bioact Mater. 2024;33:545–561. 38162513 10.1016/j.bioactmat.2023.10.032 PMC10755683

[2] Knights AJ, Redding SJ, Maerz T. Inflammation in osteoarthritis: The latest progress and ongoing challenges. Curr Opin Rheumatol. 2023;35(2):128–134. 36695054 10.1097/BOR.0000000000000923 PMC10821795

[3] Zheng L, Lu Z, Xu G, Niu X, Zhao J. Dual-targeted disease-modifying therapies for osteoarthritis. Lancet. 2024;403(10444):2591. 10.1016/S0140-6736(24)00475-6 38879250

[4] Tudorachi NB, Totu EE, Fifere A, Ardeleanu V, Mocanu V, Mircea C, Isildak I, Smilkov K, Carausu EM. The implication of reactive oxygen species and antioxidants in knee osteoarthritis. Antioxidants. 2021;10(6):985. 34205576 10.3390/antiox10060985 PMC8233827

[5] Chen H, Tu M, Liu S, Wen Y, Chen L. Dendrobine alleviates cellular senescence and osteoarthritis via the ROS/NF-κB axis. Int J Mol Sci. 2023;24(3):2365. 36768689 10.3390/ijms24032365 PMC9916903

[6] Philp AM, Davis ET, Jones SW. Developing anti-inflammatory therapeutics for patients with osteoarthritis. Rheumatology. 2017;56(6):869–881. 27498352 10.1093/rheumatology/kew278

[7] Huang Y, Liu Z, Liu C, Ju E, Zhang Y, Ren J, Qu X. Self-assembly of multi-nanozymes to mimic an intracellular antioxidant defense system. Angew Chem Int Ed Engl. 2016;55(23):6646–6650. 27098681 10.1002/anie.201600868

[8] Jena AB, Samal RR, Bhol NK, Duttaroy AK. Cellular Red-Ox system in health and disease: The latest update. Biomed Pharmacother. 2023;162: Article 114606. 36989716 10.1016/j.biopha.2023.114606

[9] Yu B, Sun W, Lin J, Fan C, Wang C, Zhang Z, Wang Y, Tang Y, Lin Y, Zhou D. Using Cu-based metal-organic framework as a comprehensive and powerful antioxidant nanozyme for efficient osteoarthritis treatment. Adv Sci. 2024;11(13): Article e2307798. 10.1002/advs.202307798 PMC10987124 38279574

[10] Yang D, Tang Y, Zhu B, Pang H, Rong X, Gao Y, Du F, Cheng C, Qiu L, Ma L. Engineering cell membrane-cloaked catalysts as multifaceted artificial peroxisomes for biomedical applications. Adv Sci. 2023;10(17): Article e2206181. 10.1002/advs.202206181 PMC10265064 37096840

[11] Joorabloo A, Liu T. Recent advances in reactive oxygen species scavenging nanomaterials for wound healing. Exploration. 2024;4(3):20230066. 38939866 10.1002/EXP.20230066 PMC11189585

[12] Wu C, Xia L, Feng W, Chen Y. MXene-mediated catalytic redox reactions for biomedical applications. ChemPlusChem. 2024;89(6): Article e202300777. 38358020 10.1002/cplu.202300777

[13] Sha M, Xu W, Fang Q, Wu Y, Gu W, Zhu C, Guo S. Metal-organic-framework-involved nanobiocatalysis for biomedical applications. Chem Catalysis. 2022;2(10):2552–2589.

[14] Signorella S, Hureau C. Bioinspired functional mimics of the manganese catalases. Coord Chem Rev. 2012;256(11-12):1229–1245.

[15] Ma P, Yang C-Y, Li C, Hu P, Yang F, Lu J, Huang Y-Y, Wu H, Wu Q, Pan Y, et al. Blow-spun Si3N4-incorporated nanofibrous dressing with antibacterial, anti-inflammatory, and angiogenic activities for chronic wound treatment. Adv Fiber Mater. 2024;6(2):543–560.

[16] Mu X, Zhang X, Liu P, Sun S, Niu J. Copper-based nanozymes: Properties and applications in biomedicine. J Inorg Mater. 2023;38(5):489–502.

[17] Xie X, Wang DP, Guo C, Liu Y, Rao Q, Lou F, Li Q, Dong Y, Li Q, Yang HB, et al. Single-atom ruthenium biomimetic enzyme for simultaneous electrochemical detection of dopamine and uric acid. Anal Chem. 2021;93(11):4916–4923. 33719390 10.1021/acs.analchem.0c05191

[18] Chen J, Kang Y, Zhang W, Zhang Z, Chen Y, Yang Y, Duan L, Li Y, Li W. Lattice-confined single-atom Fe(1) S(x) on mesoporous TiO(2) for boosting ambient electrocatalytic N(2) reduction reaction. Angew Chem Int Ed Engl. 2022;61(27): Article e202203022. 35411660 10.1002/anie.202203022

[19] Liu J, Chen L, Cui H, Zhang J, Zhang L, Su CY. Applications of metal-organic frameworks in heterogeneous supramolecular catalysis. Chem Soc Rev. 2014;43(16):6011–6061. 24871268 10.1039/c4cs00094c

[20] Yang B, Yao H, Yang J, Chen C, Shi J. Construction of a two-dimensional artificial antioxidase for nanocatalytic rheumatoid arthritis treatment. Nat Commun. 2022;13(1):1988. 35418125 10.1038/s41467-022-29735-1 PMC9008001

[21] Kumar S, Bhardwaj VK, Guleria S, Purohit R, Kumar S. Improving the catalytic efficiency and dimeric stability of Cu,Zn superoxide dismutase by combining structure-guided consensus approach with site-directed mutagenesis. Biochim Biophys Acta Bioenerg. 2022;1863(1): Article 148505. 34626596 10.1016/j.bbabio.2021.148505

[22] Im GB, Kim YG, Yoo TY, Kim YH, Kim K, Hyun J, Soh M, Hyeon T, Bhang SH. Ceria nanoparticles as copper chaperones that activate SOD1 for synergistic antioxidant therapy to treat ischemic vascular diseases. Adv Mater. 2023;35(16): Article e2208989. 36706357 10.1002/adma.202208989

[23] Abednatanzi S, Gohari Derakhshandeh P, Depauw H, Coudert FX, Vrielinck H, Van Der Voort P, Leus K. Mixed-metal metal-organic frameworks. Chem Soc Rev. 2019;48(9):2535–2565. 30989162 10.1039/c8cs00337h

[24] Yu G, Chen Y, Yang N, Zhang H, Zhang X, Geng Y, Zhao J, Chen Z, Dong C, Lin L, et al. Apoptotic bodies derived from fibroblast-like cells in subcutaneous connective tissue inhibit ferroptosis in ischaemic flaps via the miR-339-5p/KEAP1/Nrf2 axis. Adv Sci. 2024;11(24): Article e2307238. 10.1002/advs.202307238 PMC11200024 38639443

[25] Yan J, Shen M, Sui B, Lu W, Han X, Wan Q, Liu Y, Kang J, Qin W, Zhang Z, et al. Autophagic LC3(+) calcified extracellular vesicles initiate cartilage calcification in osteoarthritis. Sci Adv. 2022;8(19): Article eabn1556. 35544558 10.1126/sciadv.abn1556 PMC9094669

[26] Levine B, Kroemer G. Biological functions of autophagy genes: A disease perspective. Cell. 2019;176(1-2):11–42. 30633901 10.1016/j.cell.2018.09.048 PMC6347410

[27] Zhang H, Ni W, Yu G, Geng Y, Lou J, Qi J, Chen Y, Li F, Ye H, Ma H, et al. 3,4-Dimethoxychalcone, a caloric restriction mimetic, enhances TFEB-mediated autophagy and alleviates pyroptosis and necroptosis after spinal cord injury. Theranostics. 2023;13(2):810–832. 36632211 10.7150/thno.78370 PMC9830432

[28] Xiang L, Lou J, Zhao J, Geng Y, Zhang J, Wu Y, Zhao Y, Tao Z, Li Y, Qi J, et al. Underlying mechanism of lysosomal membrane permeabilization in CNS injury: A literature review. Mol Neurobiol. 2025;62(1):626–642. 38888836 10.1007/s12035-024-04290-6

[29] Geng Y, Lou J, Wu J, Tao Z, Yang N, Kuang J, Wu Y, Zhang J, Xiang L, Shi J, et al. NEMO-binding domain/IKKgamma inhibitory peptide alleviates neuronal pyroptosis in spinal cord injury by inhibiting ASMase-induced lysosome membrane permeabilization. Adv Sci. 2024;11(40): Article e2405759. 10.1002/advs.202405759 PMC11516130 39225315

[30] Kimura S, Noda T, Yoshimori T. Dissection of the autophagosome maturation process by a novel reporter protein, tandem fluorescent-tagged LC3. Autophagy. 2007;3(5):452–460. 17534139 10.4161/auto.4451

[31] Lorentzen KC, Prescott AR, Ganley IG. Artificial targeting of autophagy components to mitochondria reveals both conventional and unconventional mitophagy pathways. Autophagy. 2025;21(2):315–337. 39177530 10.1080/15548627.2024.2395149 PMC11760219

[32] Gu M, Jin J, Ren C, Chen X, Pan Z, Wu Y, Tian N, Sun L, Wu A, Gao W, et al. 20-Deoxyingenol alleviates osteoarthritis by activating TFEB in chondrocytes. Pharmacol Res. 2021;165: Article 105361. 33460793 10.1016/j.phrs.2020.105361

[33] Lu J, Ji ML, Zhang XJ, Shi PL, Wu H, Wang C, Im HJ. MicroRNA-218-5p as a potential target for the treatment of human osteoarthritis. Mol Ther. 2017;25(12):2676–2688. 28919376 10.1016/j.ymthe.2017.08.009 PMC5768591

[34] Sun K, Luo J, Guo J, Yao X, Jing X, Guo F. The PI3K/AKT/mTOR signaling pathway in osteoarthritis: A narrative review. Osteoarthritis Cartilage. 2020;28(4):400–409. 32081707 10.1016/j.joca.2020.02.027

[35] Xu K, He Y, Moqbel SAA, Zhou X, Wu L, Bao J. SIRT3 ameliorates osteoarthritis via regulating chondrocyte autophagy and apoptosis through the PI3K/Akt/mTOR pathway. Int J Biol Macromol. 2021;175:351–360. 33556400 10.1016/j.ijbiomac.2021.02.029

[36] Yu P, Li Y, Sun H, Zhang H, Kang H, Wang P, Xin Q, Ding C, Xie J, Li J. Mimicking antioxidases and hyaluronan synthase: A zwitterionic nanozyme for photothermal therapy of osteoarthritis. Adv Mater. 2023;35(44): Article e2303299. 37459592 10.1002/adma.202303299

[37] Jiang J, Song C, Hou X, Xu K, Ji Z, Fan L, Xi J, Zhang A. Nanozyme-driven self-assembled rhein gels for osteoarthritis therapy: Alleviating chondrocyte inflammation by reprogramming macrophages. Mater Today Bio. 2025;34: Article 102161. 10.1016/j.mtbio.2025.102161 PMC12341631 40799989

[38] Gao X, Zhang J, Gong Y, Yan L. The biomedical applications of nanozymes in orthopaedics based on regulating reactive oxygen species. J Nanobiotechnology. 2024;22(1):569. 39285458 10.1186/s12951-024-02844-3 PMC11406882

[39] Wang W, Duan J, Ma W, Xia B, Liu F, Kong Y, Li B, Zhao H, Wang L, Li K, et al. Trimanganese tetroxide nanozyme protects cartilage against degeneration by reducing oxidative stress in osteoarthritis. Adv Sci. 2023;10(17): Article e2205859. 10.1002/advs.202205859 PMC10265103 37088785

[40] Yi G, Li M, Zhou J, Li J, Song X, Li S, Liu J, Zhang H, Chen Z. Novel pH-responsive lipid nanoparticles deliver UA-mediated mitophagy and ferroptosis for osteoarthritis treatment. Mater Today Bio. 2025;32: Article 101697. 10.1016/j.mtbio.2025.101697 PMC11986606 40225130

[41] Suzuki Y, Hayashi K, Goto F, Nomura Y, Fujimoto C, Makishima M. Premature senescence is regulated by crosstalk among TFEB, the autophagy lysosomal pathway and ROS derived from damaged mitochondria in NaAsO_2_-exposed auditory cells. Cell Death Discov. 2024;10(1):382. 39191766 10.1038/s41420-024-02139-4 PMC11350138

[42] Qi Z, Yang W, Xue B, Chen T, Lu X, Zhang R, Li Z, Zhao X, Zhang Y, Han F, et al. ROS-mediated lysosomal membrane permeabilization and autophagy inhibition regulate bleomycin-induced cellular senescence. Autophagy. 2024;20(9):2000–2016. 38762757 10.1080/15548627.2024.2353548 PMC11346523

[43] Sun K, Zhang X, Hou L, Lu F, Liu H, Zheng Z, Guo Z, Xu J, Ruan Z, Hou Y, et al. TRPM2-mediated feed-forward loop promotes chondrocyte damage in osteoarthritis via calcium-cGAS-STING-NF-kappaB pathway. J Adv Res. 2025;75:213–227. 39505144 10.1016/j.jare.2024.11.007 PMC12536589

[44] Zhou Q, Ghorasaini M, Cornelis FMF, Assi R, de Roover A, Giera M, Monteagudo S, Lories RJ. Lipidomics unravels lipid changes in osteoarthritis articular cartilage. Ann Rheum Dis. 2025;84(7):1264–1276. 39894691 10.1016/j.ard.2025.01.009

[45] Ma J, Yang P, Zhou Z, Song T, Jia L, Ye X, Yan W, Sun J, Ye T, Zhu L. GYY4137-induced p65 sulfhydration protects synovial macrophages against pyroptosis by improving mitochondrial function in osteoarthritis development. J Adv Res. 2025;71:173–188. 38844123 10.1016/j.jare.2024.05.033 PMC12126715

[46] Yuan H, Shi J, Gu C, Yuan J, Huang C, Li X, Zhou K, Qi J. Akkermansia muciniphila: A next-generation gut probiotic supporting neurorepair and functional recovery. Neural Regen Res. 2025;21(8):2957–2976. 10.4103/NRR.NRR-D-25-00701 PMC13452694 41017717

