# Supplementary material for: Bimetallic Copper–Manganese Zeolitic Imidazolate Framework Nanozyme Scavenges Reactive Oxygen Species to Alleviate Osteoarthritis via Phosphoinositide 3-Kinase/Mammalian Target of Rapamycin Axis and Autophagic Flux Restoration
Source: Biomater Res. 2026 Jan 21;30:0306. doi: 10.34133/bmr.0306 (PMC12820468; doi:10.34133/bmr.0306)
Supplement: Supplementary 1 — Figs. S1 to S9 [file bmr.0306.f1.docx]

**Supplementary Material**

**Bimetallic Copper-Manganese Zeolitic Imidazolate Framework Nanozyme Scavenges Reactive Oxygen Species to Alleviate Osteoarthritis via Phosphoinositide 3-Kinase/Mammalian Target of Rapamycin Axis and Autophagic Flux Restoration**

Xiaoyu Zheng^1†^, Su Zhao^2†^, Shuming Li^3^, Yanli Wang^3^, Jiani Shi^3^, Yufei Qiu^1^, Xutong Wu^4^, Yanping Zhao^3*^_,_ Tao Jia^5*^ and Tianqi Dai^3*^

^1^Department of Anesthesiology, Harbin Medical University Cancer Hospital, Harbin 150001, China. ^2^Department of Thoracic Surgery, Harbin Medical University Cancer Hospital, Harbin 150001, China. ^3^Department of Rheumatology, The First Affiliated Hospital of Harbin Medical University, Harbin 150001, China. ^4^Harbin Medical University, Harbin 150001, China. ^5^NHC and CAMS Key Laboratory of Molecular Probe and Targeted Theranostics, Molecular Imaging Research Center (MIRC); Department of Nuclear Medicine, the Fourth Hospital of Harbin Medical University, 150028 Harbin, Heilongjiang, China

***Address Correspondence to:** [dtq19920513@163.com](mailto:dtq19920513@163.com) (T. D.); [jiatao@stu.hit.edu.cn](mailto:jiatao@stu.hit.edu.cn) (T. J.); [13303607658@163.com](mailto:13303607658@163.com) (Y. Z.)

†These authors contributed equally to this work.

‡Tianqi Dai is the paper’s lead contact.


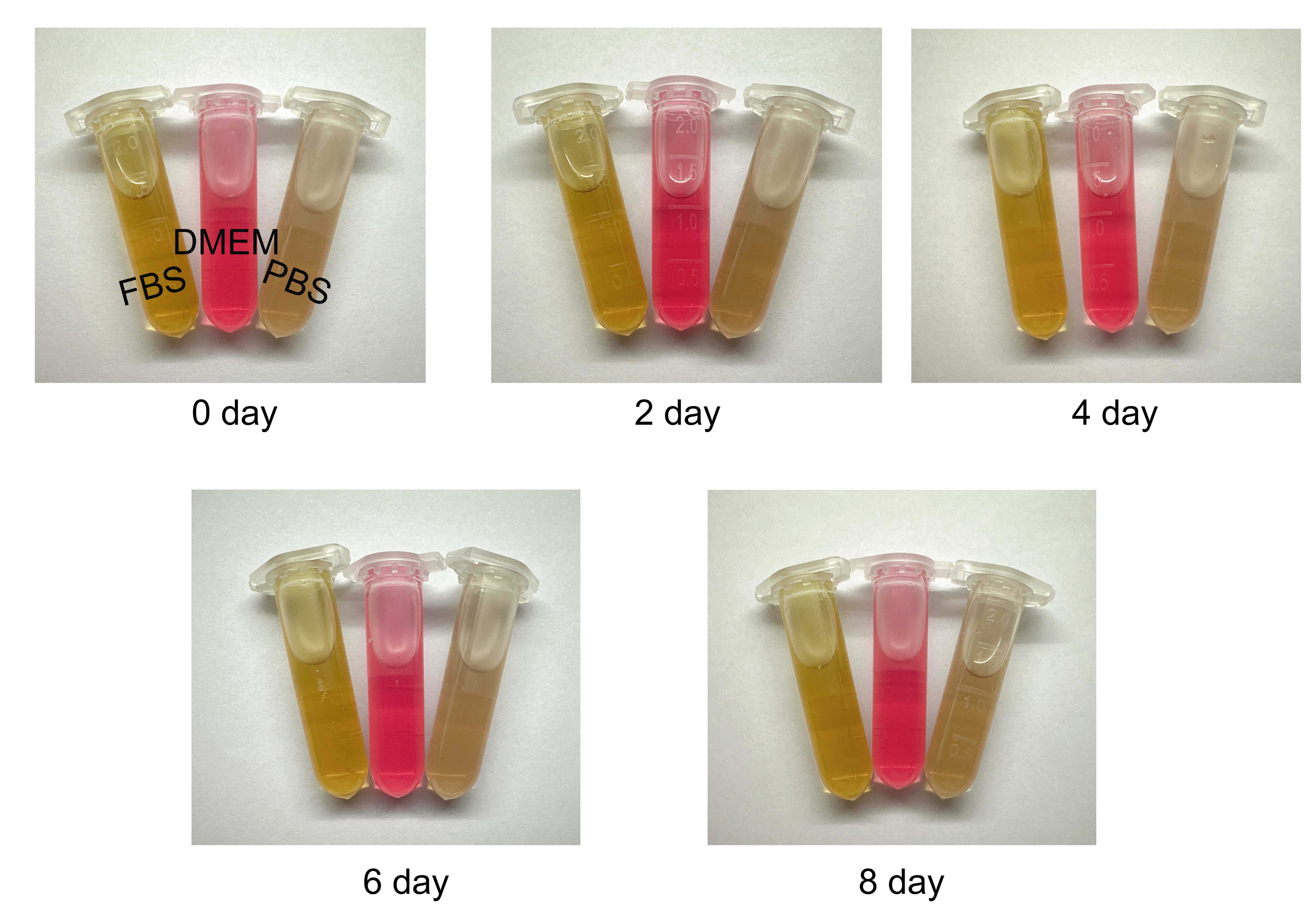


**Fig. S1.** Digital photos of the CuMn-ZIF nanozyme (100 μg/mL) taken on different days (0-8 day) in FBS, DMEM and PBS solutions.

**
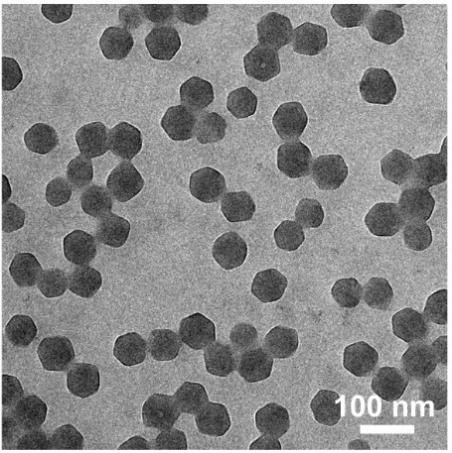
**

**Fig. S2.** TEM image of CuMn-ZIF after following treatment with ROS　(50 μmol/L of H_2_O_2_, •OH and •O_2_⁻).


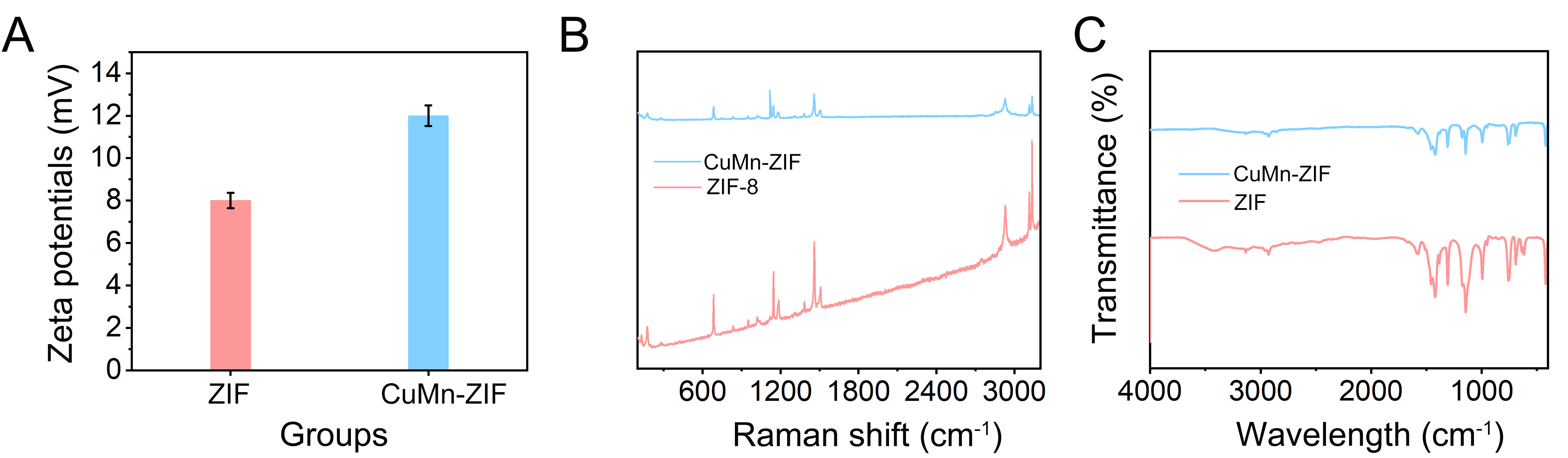


**Fig. S3.** (A) Zeta potentials, (B) Raman spectrum and (C) Fourier Transform Infrared Spectroscopy (FTIR) of ZIF and CuMn-ZIF nanozyme. Data were represented as mean ± SD (n = 3). **
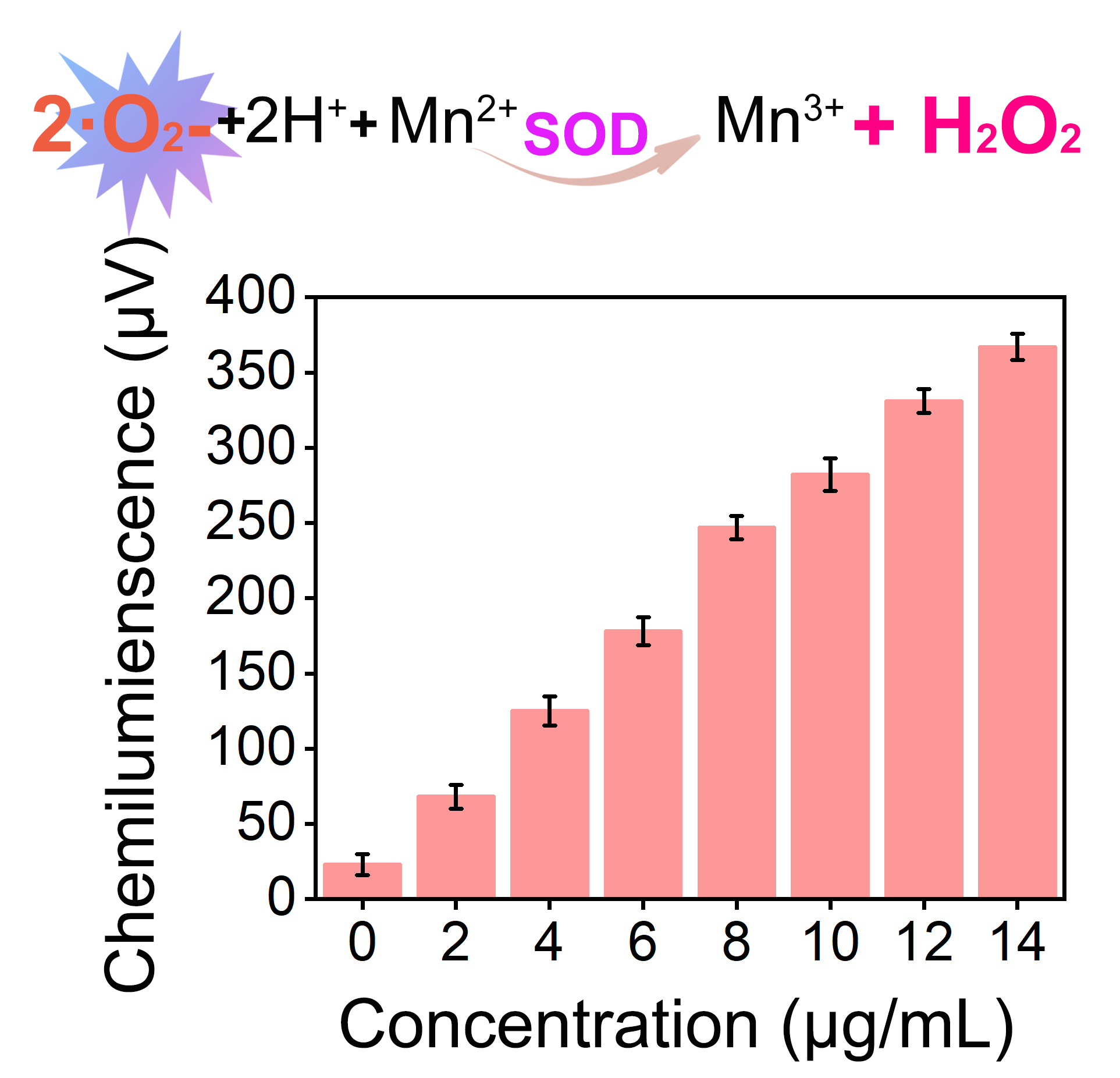
**
**Fig. S4.** Chemiluminescence profiles of luminol treated with on diverse concentrations of CuMn-ZIF nanozyme. Data were represented as mean ± SD (n = 3).

**
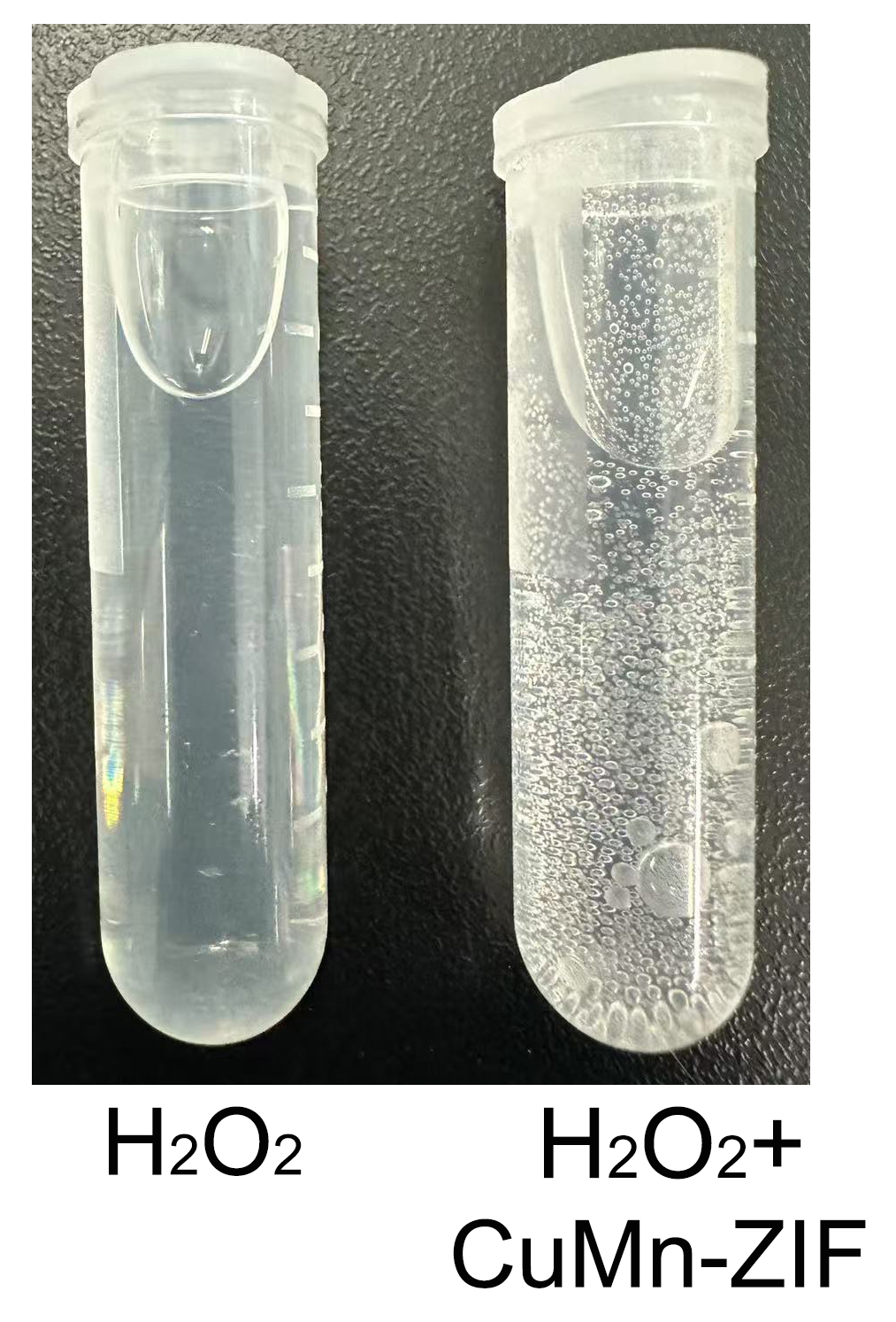
**

**Fig. S5.** Digital photography of H_2_O_2_ treatment in the presence and absence of CuMn-ZIF nanozymes.


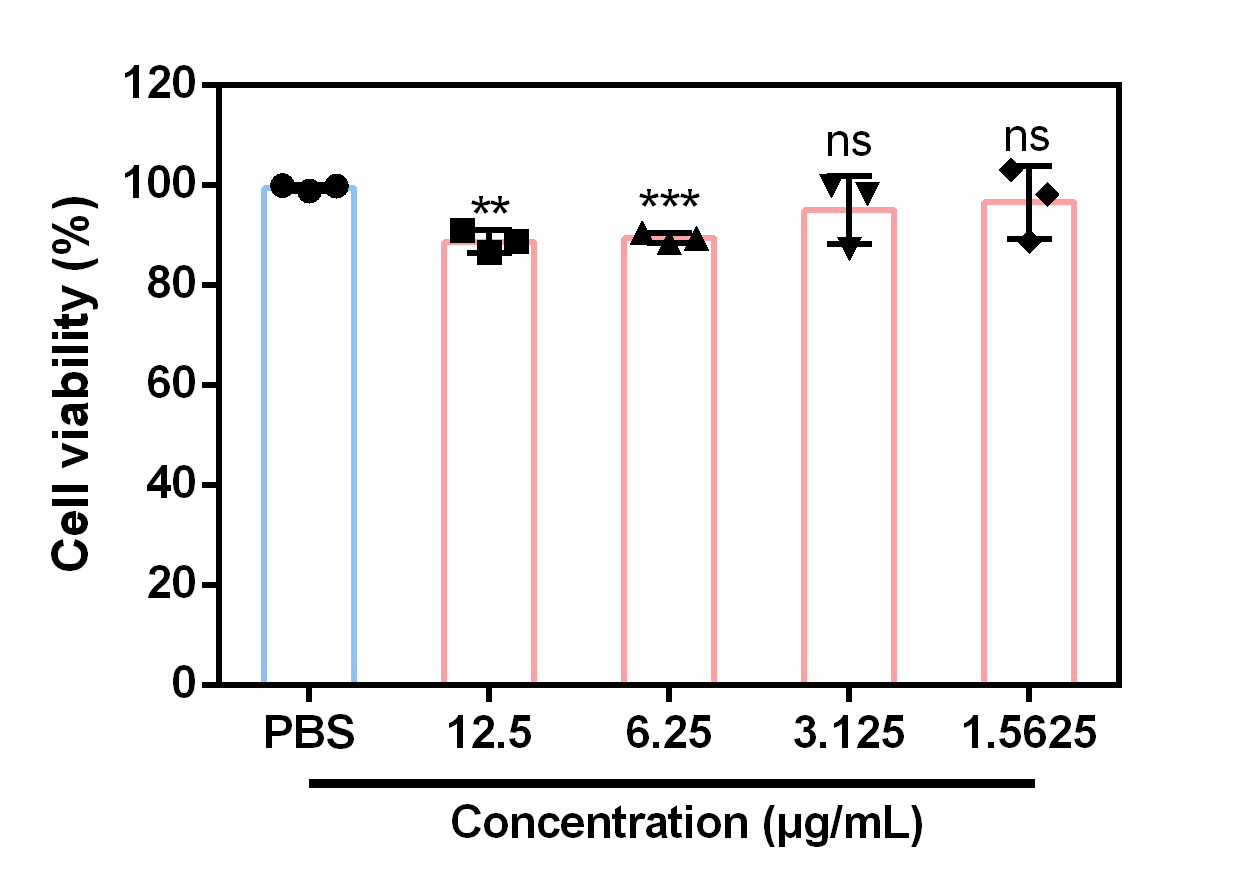


**Fig. S6.** Dose-dependent effect of CuMn-ZIF on chondrocyte viability. Viability of chondrocytes treated with increasing concentrations of CuMn-ZIF for 24 hours, as determined by the CCK-8 assay. The nanozyme maintained high cell viability across a wide concentration range, demonstrating its excellent biocompatibility. Statistical analysis was conducted using one-way ANOVA analysis. Data were represented as mean ± SD (n = 3). *****p* < 0.0001, ****p* < 0.001, ***p* < 0.01, **p* < 0.05, ns: not significant.


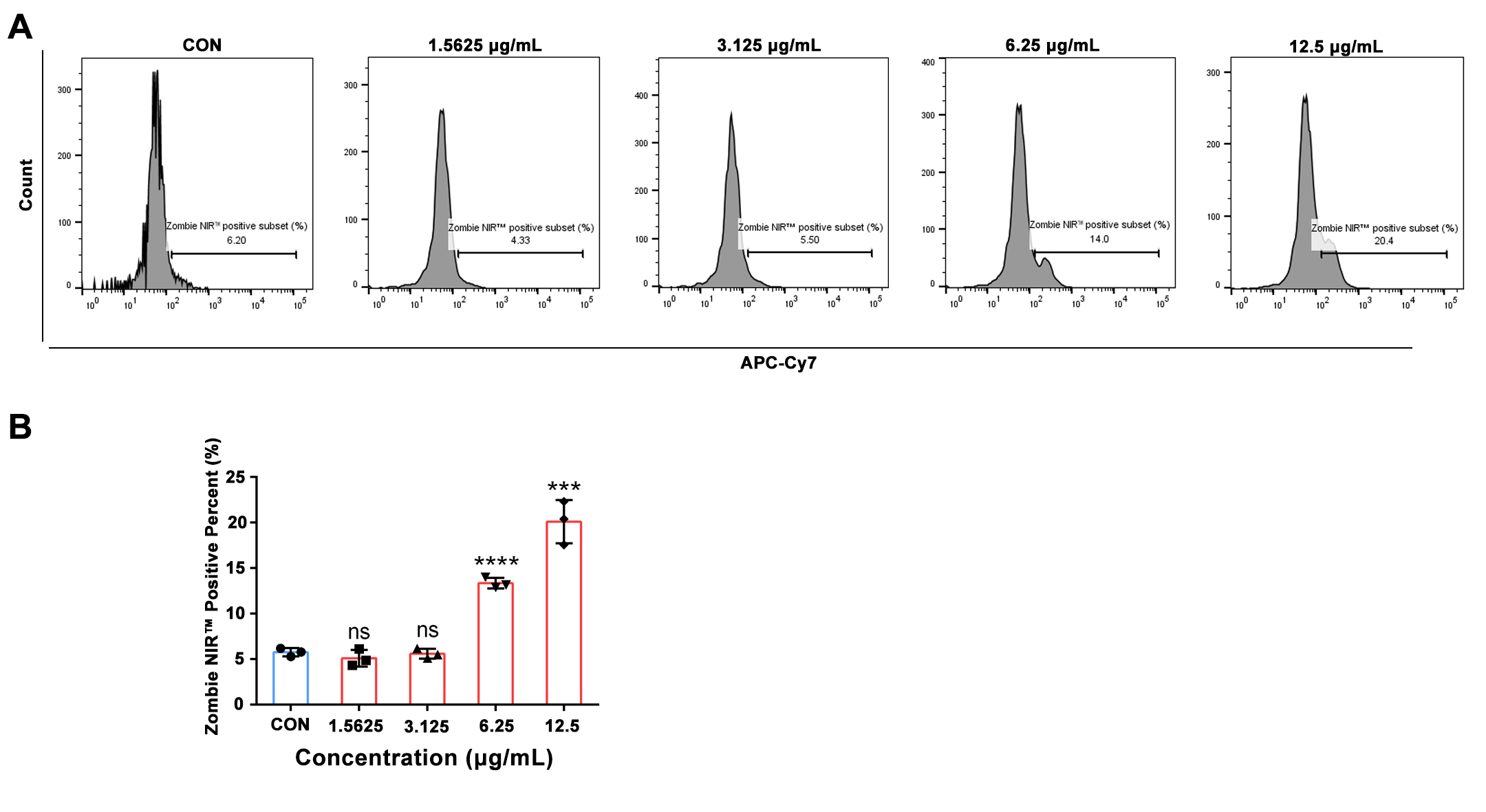


**Fig. S7.** Assessment of CuMn-ZIF cytotoxicity in chondrocytes via Zombie NIR™ staining. (A) Chondrocytes were treated with the indicated concentrations of CuMn-ZIF, and (B) quantitative analysis of cell viability was assessed by Zombie NIR™ staining followed by flow cytometry. Statistical analysis was conducted using one-way ANOVA analysis. Data were represented as mean ± SD (n = 3). *****p* < 0.0001, ****p* < 0.001, ***p* < 0.01, **p* < 0.05, ns: not significant.


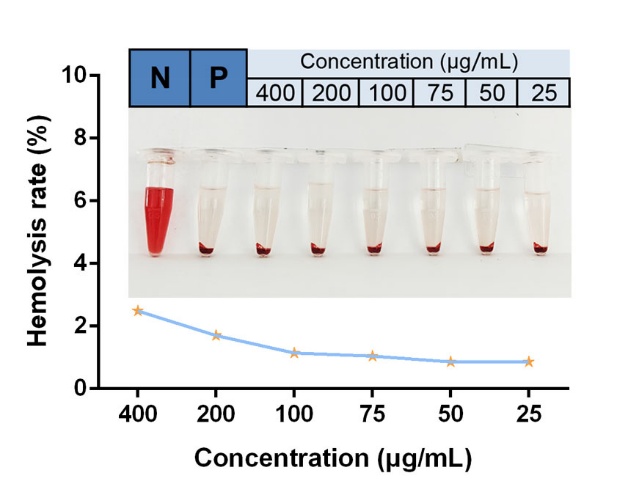


**Fig. S8.** In vitro hemocompatibility assessment of CuMn-ZIF. The experimental setup included: (1) PBS control (non-hemolytic, left tube), (2) water control (complete hemolysis, second tube), and (3) a concentration series of CuMn-ZIF suspensions for dose-dependent assessment. Statistical analysis was conducted using one-way ANOVA analysis. Data were represented as mean ± SD (n = 3). *****p* < 0.0001, ****p* < 0.001, ***p* < 0.01, **p* < 0.05, ns: not significant.


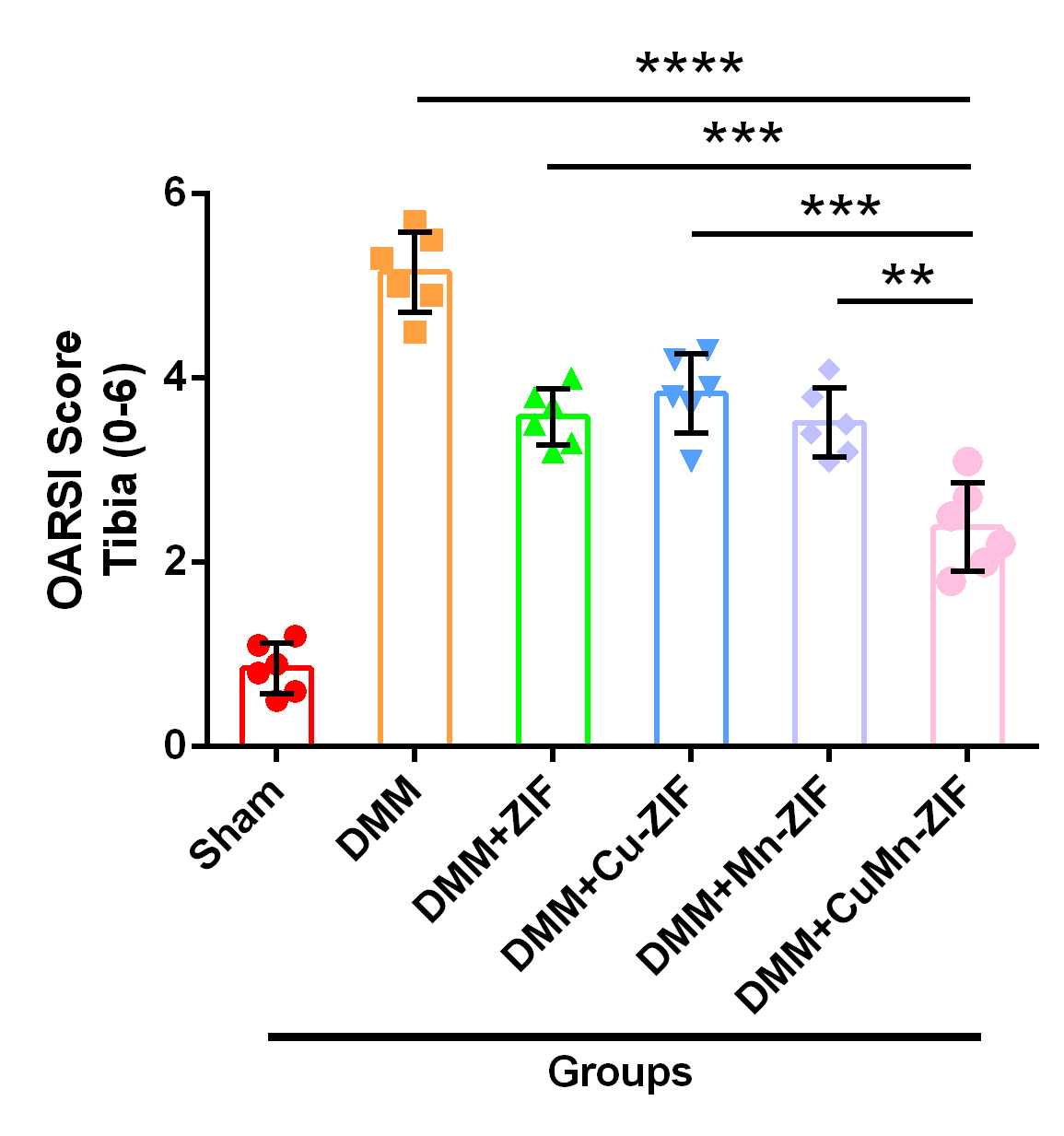


**Fig. S9.** Assessment of cartilage damage via OARSI scoring. OARSI scoring of tibial cartilage from mice subjected to DMM surgery or sham operation. Mice were treated with ZIF, Cu-ZIF, Mn-ZIF or CuMn-ZIF as indicated. Data are presented as the mean ± SD (n=6 mice per group). *P* values were calculated using one-way ANOVA with Tukey's post-hoc test. *****p* < 0.0001, ****p* < 0.001, ***p* < 0.01, or **p* < 0.05, ns: not significant.
